# Complete mitochondrial genome of the Verticillium-wilt causing plant pathogen *Verticillium nonalfalfae*

**DOI:** 10.1371/journal.pone.0148525

**Published:** 2016-02-03

**Authors:** Vid Jelen, Ronnie de Jonge, Yves Van de Peer, Branka Javornik, Jernej Jakše

**Affiliations:** 1 Biotechnical Faculty, University of Ljubljana, Ljubljana, Slovenia; 2 Department of Plant Systems Biology, VIB and Department of Plant Biotechnology and Bioinformatics, Gent University, Gent, Belgium; 3 Bioinformatics Institute Ghent, Technologiepark 927, 9052 Ghent, Belgium; 4 Department of Genetics, Genomics Research Institute, University of Pretoria, Pretoria, South Africa; Ruhr-University Bochum, GERMANY

## Abstract

*Verticillium nonalfalfae* is a fungal plant pathogen that causes wilt disease by colonizing the vascular tissues of host plants. The disease induced by hop isolates of *V*. *nonalfalfae* manifests in two different forms, ranging from mild symptoms to complete plant dieback, caused by mild and lethal pathotypes, respectively. Pathogenicity variations between the causal strains have been attributed to differences in genomic sequences and perhaps also to differences in their mitochondrial genomes. We used data from our recent Illumina NGS-based project of genome sequencing *V*. *nonalfalfae* to study the mitochondrial genomes of its different strains. The aim of the research was to prepare a *V*. *nonalfalfae* reference mitochondrial genome and to determine its phylogenetic placement in the fungal kingdom. The resulting 26,139 bp circular DNA molecule contains a full complement of the 14 "standard" fungal mitochondrial protein-coding genes of the electron transport chain and ATP synthase subunits, together with a small rRNA subunit, a large rRNA subunit, which contains ribosomal protein S3 encoded within a type IA-intron and 26 tRNAs. Phylogenetic analysis of this mitochondrial genome placed it in the *Verticillium* spp. lineage in the *Glomerellales* group, which is also supported by previous phylogenetic studies based on nuclear markers. The clustering with the closely related *Verticillium dahliae* mitochondrial genome showed a very conserved synteny and a high sequence similarity. Two distinguishing mitochondrial genome features were also found—a potential long non-coding RNA (*orf414*) contained only in the *Verticillium* spp. of the fungal kingdom, and a specific fragment length polymorphism observed only in *V*. *dahliae* and *V*. *nubilum* of all the *Verticillium* spp., thus showing potential as a species specific biomarker.

## Introduction

The *Verticillium* genus encompasses several invasive, soil-borne species of fungal plant pathogens, which infect over 400 plant species, including herbaceous annuals, perennials and even woody species, by colonizing the plant’s vascular tissues. Often the consequence of such a colonization is a wilting disease, which can be a major constraint in crop production since there are no efficient disease control measures, other than the use of resistant host varieties. *V*. *nonalfalfae (*formerly named *V*. *albo-atrum* according to [1]), among other hosts also infects hop plants, causing yellowing and wilting, adversely affecting the fitness and yield and ultimately reducing the economic value of the crop. A new *Verticillium* taxonomy, comprising ten species, has recently been introduced and is based on phylogenetic analyses of the ribosomal regions, selected sequences of protein coding genes and morphological investigations, including analysis of herbarium material and literature data [1]. The authors have also developed simplex and multiplex PCR assays for straightforward identification of *Verticillium* species [2].

*V*. *nonalfalfae* isolates from hop exhibit two types of virulence levels and are accordingly classified into 2 pathotypes—mild and lethal. The mild form is mainly influenced by environmental conditions and rarely causes complete dieback of the hop plant, which appears healthy in the following year, while the lethal form is less affected by environment and triggers rapid hop weakening followed by death of the plant. The lethal form was first discovered in UK hop production in the 40s [3], in Slovenia in the late 90s [4]and recently it was also confirmed in German hop fields [5]. The strains are not distinguishable on the morphological level, even though their virulence levels differ greatly. However, they can be distinguished by virulence tests on hop plants [4], by molecular markers [6] and by proteome level examination [7]

Due to the unique pathogenicity system described for hop *V*. *nonalfalfae* strains, they are an attractive model for virulence studies of this pathogen. We therefore carried out *de-novo* genome sequencing of mild and lethal pathotypes using next generation sequencing (NGS) Illumina technology. In such cases, sequencing projects of an eukaryotic organism can also reveal organellar genomes, in addition to the nuclear genome. This was the case with our *V*. *nonalfalfae* sequencing project, by which we discovered a mitochondrial genome. We describe here its organization and characterization.

Most mitochondrial genomes in sexual eukaryotes are uniparentally inherited, which is also true for numerous fungi, although biparental inheritance has also been reported [8]. Mitochondrial DNA lacks methylation, shows highly conserved core protein-encoding genes and is present in many copies within each cell. Because of these properties, mitochondrial gene sequences have been widely used as markers for population and species characterization [9]. Fungal mitochondrial genomes, for instance, typically encode 14 proteins related to the respiratory chain complexes, two ribosomal subunits, a distinct set of tRNAs and a variable number of free-standing open reading frames of unknown function [9].

A publically available and fully characterized mitochondrial genome from the *Verticillium* genus is that of *V*. *dahliae* (Genbank NC_008248.1, 27184 bp) [10]. It was sequenced with traditional Sanger-based methodology and was used taxonomically to characterize the species by comparing it to other known complete mitochondrial genomes from the subdivision Pezizomycotina [10]. A dideoxy-based sequencing approach is still in use for sequencing acquisition of fungal mitochondria [11–15]. However, with the general adoption of next generation sequencing (NGS) techniques, many more fungal species mitochondrial genomes have been acquired. The use of mitochondrial genomes for phylogenetic studies has become a generally well-adopted approach, and is commonly used to gain important insights into both basidio- [16–18] and asco- [19,20] mycete divisions. The most common approach for phylogenetic studies of mitochondrial sequences is to utilize a set of conserved proteins from their annotated sequences [9,21,22,23].

The increasing number of fungal mitochondrial genomes is well documented in the RefSeq-derived NCBI Organelle Genome Resource [24] database, which currently has 324 fungal mitochondrial genome entries listed (October 2015). Fungal mitochondrial genomes show a great variety of sizes, ranging from a minimum of 12,055 bp (Genbank NC_021611—*Rozella allomycis*) to a maximum of 235,849 bp (Genbank NC_021436—*Rhizoctonia solani*), with an overall average size of 50,512 bp. These size differences are mainly attributed to the numbers and lengths of intronic regions, as well as intergenic regions. For example, [25] reports that intronic regions in mitochondrial genomes can be present in different numbers and lengths, from a few bp up to 5 kb. Despite these size differences, mitochondrial genomes still predominantly contain the conserved set of protein-coding genes [26]. Other sources of fungal mitochondrial genome variation are variability in the mitochondrial gene order, presence of repeats and variable numbers of ORFs with an unknown function. This is similar in plant mitochondrial genomes, which can also have large intergenic, intronic or repetitive regions [27]. Size variability of fungal mitochondrial genomes may also be a consequence of the presence of AT-rich intergenic spacers, comprising significant portions of the mitochondrial DNA [28].

Data from our NGS genome sequencing project enabled the assembly of mitochondrial genomes of *V*. *nonalfalfae* strains from hop fields in different geographic locations (Slovenia, Germany and England) and of different pathotypes. Here we describe the completely analyzed *V*. *nonalfalfae* mitochondrial genome, which was further used for taxonomic characterization of the species. This work expanded the pool of available genomic resources for fungal mitochondrial genomes, particularly for the *Verticillium* spp. lineage and confirmed the uniformity of mitochondrial sequences between the different strains of *V*. *nonalfalfae*.

## Materials and Methods

### Data description and mitochondrial genome assembly

The NGS genomic and RNA-seq data used in this study originate from *V*. *nonalfalfae* comparative genome sequencing by our lab of six *V*. *nonalfalfae* strains isolated from hop plants, from three geographic locations (Slovenia—T2 and Rec, Germany—P15 and P55, United Kingdom– 1953 and 1985) and of two virulence levels (mild—Rec, P55 and 1953; lethal—T2, P15 and 1985). The raw genome and transcriptome sequencing data produced by the Illumina Genome Analyzer IIx (150 bp paired end) are available from the NCBI SRA archive under Bioproject PRJNA283258. The following SRA experiments for genomic data were used in the analysis: T2 (SRX1020587, SRX1020589, SRX1020590), Rec (SRX1020612, SRX1020613), P15 (SRX1021611), P55 (SRX1021624), 1953 (SRX1021626), and 1985 (SRX1021650). RNA-seq data for Slovenian strains T2 and Rec are available under SRA experiments accession numbers SRX1020629 and SRX1020679, respectively.

High molecular weight DNA of six fungal strains was obtained from germinating conidia following the protoplasts isolation procedure [29] and CTAB isolation [30], including RNAse treatment. DNA was quantified by NanoVue spectrophotometer and quality checked by agarose gel electrophoresis. Three biological replicates of Slovenian isolates T2 and Rec were grown in liquid xylem simulating media for 72 hours. RNA isolation was performed by TRIZOL reagent, isolated RNA was quantified by NanoVue spectrophotometer and integrity checked by formaldehyde gel electrophoresis. Illumina sequencing was performed at IGA Technology Services Srl (Udine, Italy) in a paired-end setup on GAIIx or HiSeq2500 sequencer.

A5 [31], Velvet [32] and CLC Genomics Workbench were used for the initial genome assemblies of the 6 *V*. *nonalfalfae* strains. Their mitochondrial genomes were identified from the assembled contigs by using the Blast+ suite [33] to align the contigs to the *V*. *dahliae* genome [10]. Only 1 contig per strain showed similarity on the nucleotide level to the published *V*. *dahliae* mitochondrial genome (NC_008248.1). Similarity between strains was investigated by remapping the sequencing reads to the assembled reference mitochondrial sequence using the ‘Map Reads to Reference’ tool and the ‘Basic Variant Detection Tool’ of the CLC Genomics package. However, all of the identified six mitochondrial contigs were non-polymorphic (100% identity), so further downstream analyses were performed on this one common mitochondrial sequence. Nucmer software [34] was used to perform global alignment between the mitochondrial genomes of *V*. *nonalfalfae* and *V*. *dahliae*.

### Annotation of the mitochondrial genome

The reference gene models were inferred with the Maker2 [35] pipeline, combining *ab-initio* gene predictor GeneMark [36], homology-based alignments of 871 V*erticillium* spp. fungal mitochondrial proteins (NCBI Entrez query: “fungi[Organism] AND verticillium AND mitochondrial”, September 2014) from the 'nr' collection of Genbank with Exonerate [37] and assembled transcripts from RNA-Seq data. The *Verticillium* transcripts were prepared by mapping RNA-Seq data to the mitochondrial genome with Tophat [38] and assembling transcripts with Cufflinks [39]. Assembled transcripts were screened for candidate coding regions and finalized with TransDecoder [40].

tRNAs were annotated with tRNAscan-SE (v1.21) [41] and their secondary structures were predicted with ARWEN [42]. Secondary structures were visualized with the VARNA [43] visualization java applet. The mitochondrial genome was additionally examined for tRNAs, rRNAs and introns with RNAweasel [44] and MFannot [45]. The codon usage of the mitochondrial genome was calculated with the 'cusp' program of the EMBOSS suite [46]. The programs were run with default parameters, the only exception being setting the genetic code to mitochondrial where applicable.

Initial structural annotations and BAM file tracks of RNA-Seq mapping were loaded onto a local installation of the WebApollo [47] server for visualization and manual curation. After curation, the final dataset was functionally annotated with the Blast2GO suite [48].

BLAST characterization of orf414 was performed using the on-line BLAST server at NCBI using BLASTn searches against 9 nucleotide databases (nucleotide collection (nr/nt), reference RNA sequences (refseq_rna), reference genomic sequences (refseq_genomic), refseq representative genomes (refseq_representative_genomes), NCBI Genomes (chromsome), expressed sequence tags (est), genomic survey sequences (gss), high throughput genomic sequences (HTGS), transcriptome Shotgun Assembly (TSA) sequences (tsa_all)) and a BLASTX search against non-redundant protein sequences (nr).

The graphic presentation of the annotated mitochondrial genome was prepared with Circos [49].

The complete sequence of the mitochondrial genome of *V*. *nonalfalfae* has been deposited in Genbank under accession no. KR704425.

### Phylogenetic analysis

We selected a set of publicly available fully annotated fungal mitochondrial genomes from a broad taxonomic spectrum of fungal species, including 15 pathogenic fungi from the Pezizomycotina group and 3 yeasts, both of which represented the group of ascomycetes, and a single basidiomycete pathogen (Table 1) as an outgroup, to conduct the phylogenetic analysis and to determine the placement of *V*. *nonalfalfae* among them. Amino acid sequences of the 14 conserved mitochondrial proteins [9] from these species, including four Cytochrome c oxidase subunits (cox1, cox2, cox3 and cob), three ATP synthase subunits (atp6, atp8 and atp9) and seven NADH dehydrogenase subunits (nad1, nad2, nad3, nad4, nad4L, nad5 and nad6) were aligned using Muscle [50] and trimmed with trimAl [51] to remove badly aligned amino acid sequences. The alignments were then concatenated and used to construct a phylogenetic tree. RAxML’s [52] automated model selection was used to determine the best protein model in terms of likelihood on a fixed, reasonable tree, along with the GAMMA model of rate heterogeneity. The final maximum likelihood phylogenetic tree was made by running RAxML's rapid bootstrap algorithm with 100 approximations and a subsequent maximum likelihood search. ETE [53] was used to visualize the resulting trees.

**Table 1:** Species included in the phylogenetic analysis.

Genbank ID	Taxonomy	Name	Genome size (bp)	Average GC content	# of tRNAs
NC_023540.1	*Glomerellales*	*Colletotrichum lindemuthianum*	36,957	30.88%	28
NC_001329.3	*Sordariales*	*Podospora anserina*	100,314	30.06%	27
NC_023127.1	*Helotiales*	*Rhynchosporium orthosporum*	49,539	28.80%	29
NC_008068.1	*Hypocreales*	*Metarhizium anisopliae*	24,673	28.40%	24
KC683708.1	*Sordariales*	*Neurospora crassa*	64,840	36.13%	28
NC_010222.1	*Capnodiales*	*Zymoseptoria tritici*	43,964	31.94%	27
NC_004514.1	*Hypocreales*	*Lecanicillium muscarium*	24,499	27.15%	25
NC_016680.1	*Hypocreales*	*Fusarium solani*	62,978	28.88%	25
NC_025200.1	*Helotiales*	*Sclerotinia borealis*	203,051	32.01%	31
NC_017930.1	*Hypocreales*	*Fusarium oxysporum*	34,477	30.98%	25
NC_007445.1	*Eurotiales*	*Aspergillus niger*	31,103	26.90%	25
NC_001326.1	*Schizosaccharomycetales*	*Schizosaccharomyces pombe*	19,431	30.09%	25
NC_009493.1	*Hypocreales*	*Fusarium graminearum*	95,676	31.84%	28
NC_008248.1	*Glomerellales*	*Verticillium dahliae*	27,184	27.32%	25
NC_023268.1	*Hypocreales*	*Acremonium chrysogenum*	27,266	26.54%	26
NC_001224.1	*Saccharomycetales*	*Saccharomyces cerevisiae*	85,779	17.11%	24
NC_018046.1	*Saccharomycetales*	*Candida albicans*	33,928	31.74%	24
NC_022835.1	*Hypocreales*	*Metacordyceps chlamydosporia*	25,615	28.28%	22
NC_008368.1	*Ustilaginales*	*Ustilago maydis*	56,814	31.20%	23
KR704425	*Glomerellales*	*Verticillium nonalfalfae*	26,139	26.92%	26

### RT-qPCR of long non-coding RNA (*orf414)*

Fungal strains T2 and Rec were maintained and grown on 1/2 Czapek Dox agar plates at room temperature. Fungal mycelium was harvested from cultured plates and RNA of three biological replicates (50 mg) for each strain was isolated using a spin column procedure employing a Spectrum Plant Total RNA Extraction Kit (Sigma-Aldrich). Isolation followed the manufacturer’s protocol, including on column DNAse digestion (Sigma-Aldrich). The RNA concentrations and A260/A280 ratios were measured by a NanoVue Spectrophotometer (GE Healthcare). Quantified RNA samples were stored at -80°C.

One μg of each RNA sample was reverse-transcribed to cDNA using a High Capacity cDNA Reverse Transcription Kit (Applied Biosystems, Foster City, USA), employing random hexamer primers. Reverse-transcribed samples were stored at −20°C. Primers ORF414-FOR 5’-ATCCGAGGGAGATGAGACTTCA-3’ and ORF414-REV 5’-TCACCTGATCTTTCTTCAACTTCAA-3’ for *orf414* were constructed using Primer Express version 3.0 (Applied Biosystems) with default parameter settings. As a housekeeping reference, the ubiquitin gene (UBQ) was amplified with primers UBQ-FOR 5’-GACTCGACCTCAAGGGTGAT-3’ and UBQ-REV 5’-GTCTTCGTGGTGGTATGCAG-3. The housekeeping reference was selected on the basis of a previous study [54], in which the ubiquitin gene was selected as the most stably expressed in the analysis of *V*. *dahliae*. A qPCR reaction mixture was prepared in a 10 μl reaction volume containing 5 μl of FAST SYBR Green PCR Master Mix (Applied Biosystems), 2 μl of cDNA template and 0.6 μM of each specific primer. FAST Amplification was performed in the ABI PRISM 7500 Fast Sequence Detection System (Applied Biosystems, Foster City, USA) with the following cycling program: 95°C for 20 s, 40 cycles at 95°C for 3 s, followed by 60°C for 30 s, followed by melting curve analysis to confirm the amplification of a single PCR product. All samples were amplified in technical triplicates and the mean cycle threshold (Ct) value was calculated to investigate the expression levels of the analyzed *orf414*. A t-test for differential expression analysis between the mild and lethal strains was conducted with R v3.0.2.

### *Verticillium* mitochondrion specific length polymorphisms

The assembled *V*. *nonalfalfae* mitochondrial genome was aligned with the *V*. *dahliae* mitochondrial genome to analyze their similarities using Nucmer [55] pairwise alignment. Furthermore, Illumina sequencing reads of 6 *V*. *nonalfalfae* strains were mapped to the reference *V*. *dahliae* mitochondrial genome using the ‘Map reads to reference’ tool of the CLC Genomics Workbench. A *V*. *dahliae* lineage specific region was found, which was further PCR amplified in different isolates of the *Verticillium* spp. A conserved primer pair mth-for 5’-CCTCACGCTTTTGTAAGTTTACCT-3’ and mth-rev 5’-AATTCAAACTCGTTAATACATAGCA-3’ was constructed using PRIMER3 online software.

Primers were used for amplification of a *Verticillium* species collection (96 samples), consisting of *V*. *albo-atrum* (6 samples), *V*. *alfalfae* (5 samples), *V*. *dahliae* (26 samples), *V*. *isaacii* (3 samples), *V*. *nonalfalfae* (49 samples), *V*. *nigrescens* (2 samples), *V*. *tricorpus* (2 samples), *V*. *longisporum* (2 samples), and *V*. *nubilum* (1 sample) (S1 Table). PCR reactions were performed in a volume of 20 μl containing fungal DNA (1:100 diluted CTAB isolation), 1x supplied PCR buffer, 2 mM MgCl_2_, 0.8 mM dNTP solution and 0.5 U of *Taq* DNA polymerase (Kapa Biosystems). Amplification cycling conditions were as follows: 95°C for 5 min, followed by 5 cycles of 95°C 30 sec, 65°C 30 sec (at each cycle the temperature was lowered by 1°C) and 72°C 1 min 30 sec, followed by 25 cycles with same time and temperature profiles, except that the annealing temperature was 55°C. Amplification products were resolved on 1.2% agarose gel.

## Results

The mitochondrial genome of *V*. *nonalfalfae* presented in this work is a circular-mapping DNA molecule of 26,139 bp in size, with a mean GC content of 26.92%. The three assemblers produced the same mitochondrial sequence for the 6 paired-end NGS data sets. Moreover, remapping the NGS reads back to the mitochondrial genome and subsequent SNP analysis did not indicate any polymorphic regions, despite high coverage (average between 499–10,688x) achieved for all 6 data sets (Table 2). It is comparable in size to the related *V*. *dahliae* mitochondrial genome [10], published on Genbank (NC_008248.1) with a length of 27,184 bp and mean %GC content of 27,32%. A sequence-to-sequence Nucmer alignment of their mitochondrial genomes shows a 98.15% sequence identity with 99.35% aligned bases of *V*. *nonalfalfae* mitochondria to *V*. *dahliae*.

**Table 2:** Mapping results for mitochondrial sequence for 6 NGS data set used for assembly.

Item/Strain	Rec	T2	1953	1985	P55	P15
Total read count	97,295	2,040,416	203,690	761,897	250,232	351,890
Reads in pairs	84,816	1,825,642	191,878	703,388	236,896	321,930
Total read length (bp)	13,039,773	279,682,422	27,823,727	106,099,232	34,619,109	48,297,310
Minimum coverage (X)	7	65	9	42	17	12
Maximum coverage (X)	1,028	18,987	2,467	8,243	2,853	4,016
Average coverage (X)	499	10,699	1,064	4,059	1,324	1,847

### Annotation results and codon usage

**Fig 1:**
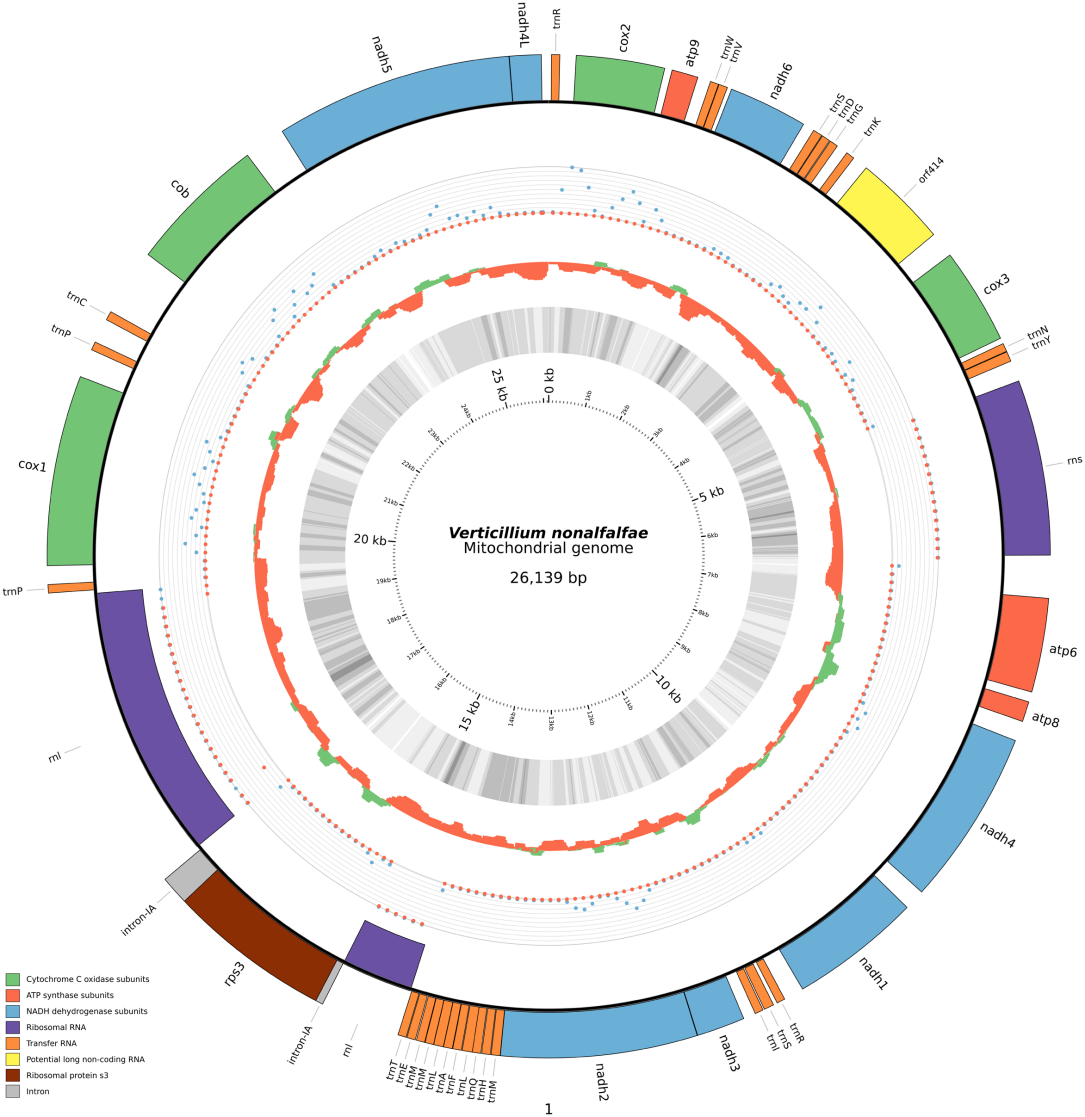
Genetic map of the *Verticillium nonalfalfae* mtDNA. The concentric circles from the outside inwards represent different tracks. The outermost track represents the coding features of the *V*. *nonalfalfae* mitochondrial genome. The direction of the highlights (inward, outward) represents the strand in which the feature is present. The next track represents read counts of RNA-Seq mapping from 2 different pathotypes (mild-blue, lethal-red) of *V*. *nonalfalfa*e. The tracks are made of Bowtie2 [56] mapped RNA-Seq reads and shown as counts per 100bp bins. The counts were normalized with the DESeq method [57]. Because of rRNA counts overwhelming the remaining expression profiles, the count number on the graph was capped below 17.5% of the top count profiles (a non-capped graph would show only rRNA expressed), in order to enable visualization of low-expressed regions. Following this track are GC-skew and GC-content tracks, respectively. GC-skew [(G-C)/(G+C)] reflects the relative number of cytosine to guanine and is often used to describe the strand-specific bias of a nucleotide composition. The GC-skew track is shown as a histogram of 250bp sliding windows with calculated gc-skew coefficients. Green regions represent windows for which the coefficient is larger than 0 and red regions windows for which the coefficient is smaller than 0. The neighbouring grayscale heatmap of the GC-content track represents 100bp sliding windows with calculated gc-contents. Regions in the GC content heatmap are shaded in gray, where darker gray represents higher gc-content and lighter gray represents lower gc-content. The two tracks show a similar pattern to other Sordariomycetes and were also used to scan for anomalies in GC content, which could indicate the introduction of heterologous DNA. No anomalies indicating such an event were detected. The cumulative GC skew analysis was also used to try and find the origins of replication and termination of replication loci (data not shown) but we could not determine them with this analysis.

The mitochondrial genome of *V*. *nonalfalfae* harbors the whole complement of 14 conserved mitochondrial protein-coding genes, namely the subunits of the electron transport chain of complex I (*nad1*, *nad2*, *nad3*, *nad4*, *nad4L*, *nad5* and *nad6*), complex III (*cox1*, *cox2*, *cox3*), complex IV (*cob*) and the ATP synthase subunits (*atp6*, *atp8* and *atp9*). Other features also present in the assembled mitochondrial genome are: a small rRNA subunit, a large rRNA subunit containing ribosomal protein S3 encoded within a type IA-intron, 26 tRNAs and a potential long non-coding RNA, designated *orf414* (Fig 1).

All mitochondrial genes are predicted to be encoded on the same strand, with the exception of the long rRNA subunit, which is encoded on the opposite strand (Fig 1, Tables 3 and 4).

**Table 3:** Gene features of the *Verticillium nonalfalfae* mitochondrial genome.

	Start position	Stop position	Length	Strand	Start Codon	Stop Codon
*cox1*	19528	21129	1602	-	AUG	UAA
*cox2*	228	971	744	-	AUG	UAA
*cox3*	3855	4664	810	-	AUA	UAA
*cob*	22290	23459	1170	-	AUG	UAA
*nad1*	9760	10863	1104	-	AUG	UAA
*nad2*	11820	13472	1653	-	AUG	UAA
*nad3*	11409	11816	408	-	AUG	UAA
*nad4*	8100	9584	1485	-	AUG	UAA
*nad4L*	25812	26078	267	-	AUG	UAA
*nad5*	23809	25809	2001	-	AUG	UAA
*nad6*	1552	2217	666	-	AUG	UAG
*atp6*	6884	7675	792	-	AUG	UAA
*atp8*	7760	7930	171	-	AUG	UAA
*atp9*	1034	1255	222	-	AUG	UAA
*rps3*	15079	16452	1374	-	AUA	UAA

**Table 4:** rRNA and *orf414* features of the *Verticillium nonalfalfae* mitochondrial genome.

	Start position	Stop position	Length	Strand
rns	5049	6525	1477	-
rnl	14354	15014	660	+
rnl	16734	19266	2533	+
orf414	2850	3638	789	-

Putative secondary structures of the tRNA molecules were also determined. It was shown that all of them can fold into a common cloverleaf structure made out of the acceptor stem, D-loop, anticodon loop, anticodon and TψC loop. Five of the tRNA molecules even have an additional variable loop: tRNA-Ser(GCU), tRNA-Tyr(GUA), tRNA-Ser(UGA), tRNA-Leu(UAG), tRNA-Ser(UAA). Their graphic depictions are presented in S1 Fig.

**Fig 2:**
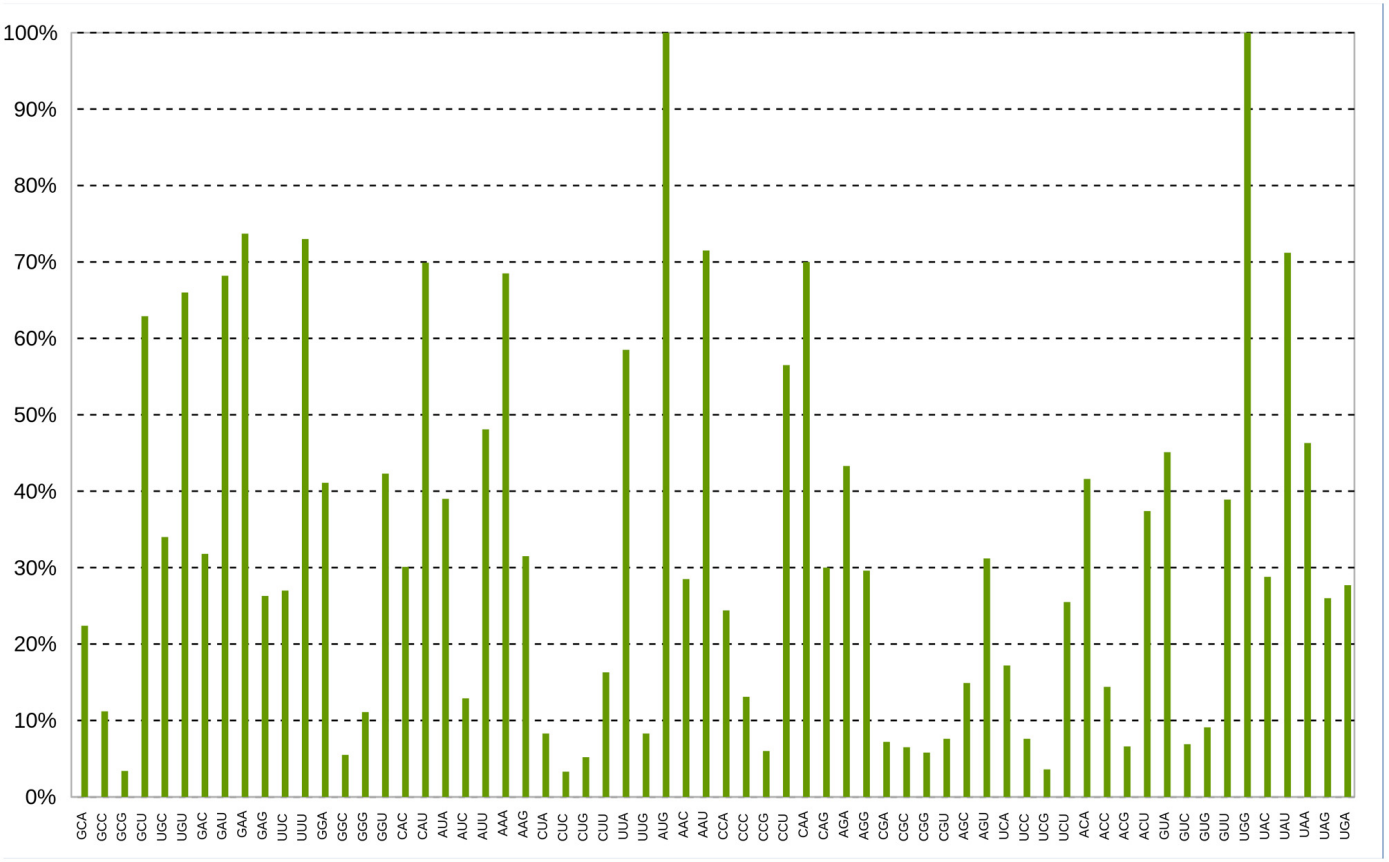
Column diagram of the *Verticillium nonalfalfae* mtDNA codon usage. The diagram represents the codons (x-axis) and percentages of their occurrence (y-axis) in the *V*. *nonalfalfae* mitochondrial genome.

The mitochondrial genome was screened for codon usage (Fig 2, S1 Table) and the genes were analyzed with respect to their start and stop codons (Table 3). Among the predicted genes, the 'AUG' initiation codon was mostly prevalent, with only the genes *cox3* and *rps3* using the alternative 'AUA' initiation codon. The most frequent stop codon was 'UAA', followed by two alternative stop codons exhibiting similar frequency. The 26 tRNA genes (Table 5) coded for 20 of the 22 common amino acids (excluding Ornithine and Selenocysteine), with the anti-codons ACG (Arginine), GCU (Serine), CAU (Methionine) and UAG/UAA (Leucine) being present in more than 1 copy. In view of the described annotation information, only 16.52% of the mitochondrial genome is represented by intergenic regions.

**Table 5:** tRNA features of the *Verticillium nonalfalfae* mitochondrial genome.

tRNA Type	Anti Codon	Start position	Stop position	Strand
Arginine	ACG	22	92	-
Tryptophan	UCA	1366	1437	-
Valine	UAC	1444	1515	-
Serine	GCU	2315	2394	-
Asparagine	GUC	2400	2472	-
Glycine	UCC	2480	2550	-
Lysine	UUU	2647	2719	-
Asparagine	GUU	4713	4783	-
Tyrosine	GUA	4788	4872	-
Arginine	UCU	11033	11103	-
Serine	UGA	11137	11221	-
Isoleucine	GAU	11234	11305	-
Methionine	CAU	13477	13549	-
Histidine	GUG	13556	13629	-
Glutamine	UUG	13636	13708	-
Leucine	UAG	13710	13792	-
Phenylalanine	GAA	13799	13871	-
Alanine	UGC	13878	13949	-
Leucine	UAA	13951	14033	-
Methionine	CAU	14035	14107	-
Methionine	CAU	14111	14181	-
Glutamate	UUC	14191	14263	-
Threonine	UGU	14270	14340	-
Proline	UGG	19299	19370	-
Proline	UGG	21370	21441	-
Cysteine	GCA	21648	21724	-

### Characterization and expression profile of the long non-coding RNA—*orf414*

BLASTn and BLASTx algorithms were used for characterizing the *orf414* sequence (2850..3638, length 789 bp) against 10 NCBI databases. Nucleotide comparisons revealed that this particular region is specific to the *Verticillium* lineage, since significant hits (e>10^−5^, >50% of query) were only found in *V*. *dahliae* sequences. Moreover, the majority of them were of *V*. *dahliae* mitochondrial origin, with 96% identity and 100% query coverage, with the exception of the chr2 genomic sequence in the nt database of JR2 *V*. *dahliae* assembly [58] which had 93% identity and 88% of query coverage. Only one significant similarity to other species was found in the EST division, with cDNA clone of *Phytophtora infestans*, which showed 95% identity over 56% of the *orf414* sequence (S1 File). A comparison with the protein database (BLASTx) did not reveal any significant hits.

**Fig 3:**
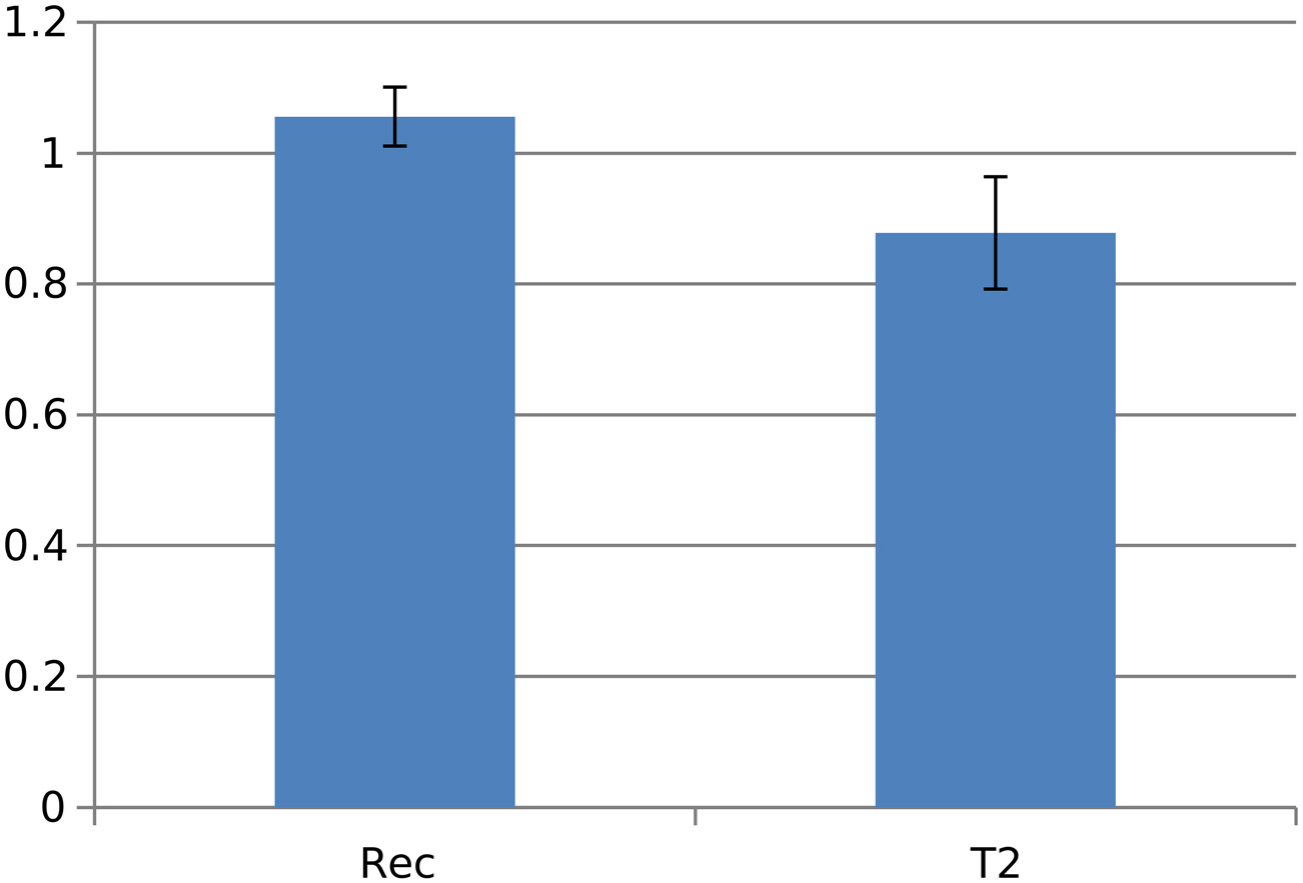
RT-qPCR analysis of *orf414*. Expression analysis of th*e* potential long non-coding RN*A orf414* in two different strains of *V*. *nonalfalfa*e (Rec = Slovenian mild strain and T2 = Slovenian lethal strain). Expression values were normalized with *ubiquitin (ub*q) as a housekeeping reference. Bars indicate standard errors of three biological replicates.

We performed a RT-qPCR analysis to confirm the *in vivo* expression of *orf414* in two Slovenian fungal strains of different pathotypes (Rec and T2) using three biological replicates. The expression of *orf414* in the mild strain was 1.2 fold higher than that in the lethal strain (Fig 3). The expression values were normalized with *ubiquitin (ubq)* as a housekeeping reference and were compared between strains with an unpaired t-test. The mean difference in expression levels of *orf414* between the mild strain (mean = 0.078, sd = 0.061, se = 0.043) and the lethal strain (mean = -0.193, sd = 0.146, se = 0.073) was evaluated with a p-value of 0.07373 and a 95% confidence interval of [-0.041, 0.583], from which we conclude that the expression of orf414 between strains is of similar levels.

### Phylogeny results

In order to infer phylogenetic relationships between the fungal species, a maximum likelihood approach was used. Concatenated amino acid sequences of conserved proteins from the 20 fungal species (Table 1) were used to perform the phylogenetic analysis.

**Fig 4:**
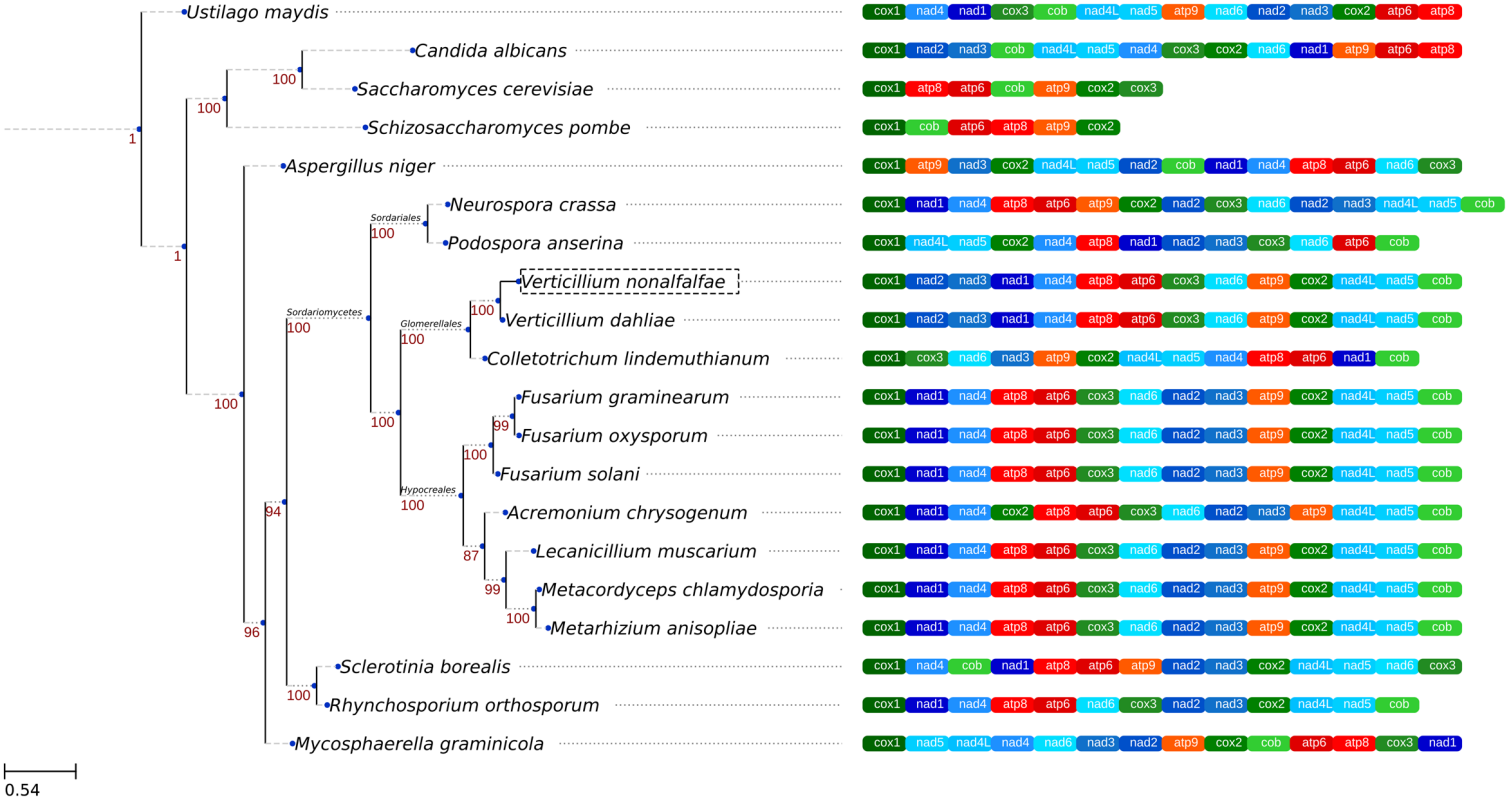
Maximum likelihood phylogenetic tree of 20 mitochondrial genomes, based on a conserved set of proteins. The tree was inferred from an alignment of amino-acid sequences of conserved mitochondrial proteins with 3093 distinct alignment positions and 100 rapid bootstrap inferences. The gamma model of rate heterogeneity and a maximum likelihood estimate of the alpha-parameter were used to prepare the final tree. Numbers above tree nodes represent the bootstrap support values. Next to the tree is a graphical presentation of an alignment of mitochondrial protein-coding genes and their order in the represented species. The conserved set of 14 protein-encoding genes is present in most of the species, with the exception of *S*. *cerevisiae* and *S*. *pombe* yeasts, which lack the NADH dehydrogenase family of genes, *C*. *lindemuthianum*, which lacks the *nad2* gene, *N*. *crassa*, which has two copies of the *nad2* gene and *R*. *orthosporum* and *P*. *anserina*, which lack the *atp9* gene. The phylogenetic tree shows *V*. *nonalfalfae* joined with *V*. *dahliae* and *C*. *lindemuthianum* in a cluster corresponding to the established group of fungi called *Glomerellales*. *C*. *lindemuthianum* can be seen to have a very different gene order compared to the other two members of its group. The *Hypocreales* and *Sordariales* groups of the Sordariomycetes can also be seen in the tree. *A*. *chrysogenum* of the *Hypocreales* group contains a translocation of the *cox2* gene, which distinguishes it from the rest of the members of its group. The *Glomerellales* and *Hypocreales* groups show a high degree of synteny within their respective groups and differ only by a translocation of the *nad2*-*nad3* gene cluster.

The resulting phylogenetic tree (Fig 4) shows high bootstrap support for the individual groups. Since mitochondria within these species are considered to be circular molecules, the alignment start position was arbitrarily decided as the *cox1* gene, which all species in this study possess.

The conserved set of 14 mitochondrial protein-encoding genes is present in most of the species used in this analysis. The exceptions are *S*. *cerevisia*e and *S*. *pombe* yeasts, which lack the NADH dehydrogenase family of genes, *C*. *lindemuthianum*, which lacks the *nad2* gene, *N*. *crassa*, which has two copies of the *nad2* gene and *R*. *orthosporum* and *P*. *anserina*, which lack the *atp*9 gene.

The analysis yielded 3 clades, which correspond to the *Glomerellales*, *Hypocreales* and *Sordariales* groups of fungi within the Sordariomycetes family. Another clade, consisting of *S*. *borealis* and *R*. *orthosporum*, corresponds to the *Helotiales* group. The yeasts (*S*. *cerevisiae*, *S*. *pombe*, *C*. *albicans*) clustered together and *U*. *maydis* is shown as an outgroup. *V*. *nonalfalfae* was positioned in the *Glomerellales* group, together with *C*. *lindemuthianum* and the closely related *V*. *dahliae*.

### Analysis of the *Verticillium dahliae* specific length polymorphism

When aligning the *V*. *nonalfalfae* and *V*. *dahliae* mitochondrial genomes, a *V*. *dahliae-*specific region (1,221 bp long) not present in *V*. *nonalfalfae* was revealed between the gene *coxI* and a tRNA coding for proline. Mapping the mitochondrial sequencing reads of our *V*. *nonalfalfae* strains to the *V*. *dahliae* mitochondrial genome also supported this finding (S2 Fig). We further analyzed the region with PCR amplification in 96 different *Verticillium* spp. isolates (S2 Table). The primers were designed in the conserved regions of the *V*. *dahliae* and V. *nonalfalfae* region with expected amplicon sizes of 1400 bp (*V*. *dahliae*) and 400 bp (*V*. *nonalfalfae*). Amplification of the region was successful in 22 out of 26 *V*. *dahliae* isolates showing a longer amplicon and in 44 out of 49 analyzed *V*. *nonalfalfae* species with a shorter amplicon. The longer amplicon was also characteristic of *V*. *nubilum* species, while the 400 bp amplicon was also amplified in *V*. *alfalfae* and *V*. *longisporum*. Amplification in species *V*. *albo-atrum*, *V*. *isaacii*, *V*. *nigrescens* and *V*. *tricorpus* did not yield any fragment.

## Discussion

The assembled mitochondrial contigs of six unique *V*. *nonalfalfae* strains were compared to reveal a possible pattern of variation. A similar approach was used by [59] to distinguish among 18 strains of *Lachancea kluyveri*, which had been isolated from various geographical locations and ecological niches. They found great diversity in the intergenic regions, with variants and indels, and also highly conserved coding regions in the mitochondrial genomes. However, in our case, the mitochondrial genomes did not reveal any variation. Assemblies of 3 different assemblers showed that their nucleotide sequences are the same. Re-mapping analysis further confirmed that no variants exist in the sets of NGS sequencing data for the six strains analyzed.

Another more frequent way of comparing mitochondrial genomes is inter-species, rather than an intra-species approach [13,26,27,56,60]. An analysis of two related soybean rust pathogens based on their mitochondrial genomes is such an example [14]. Mitochondrial genomes of *Phakopsora pachyrhizi* and *Phakopsora meibomiae* were sequenced, had their genes annotated and were phylogenetically compared to other members of *Basidiomycota* for taxonomical characterization. The results showed that the order of protein-coding genes and tRNA is identical in the two *Phakopsora* species and all genes are transcribed from the same DNA strand clockwise, while the sizes of their mitochondrial genomes are also quite similar (31,825bp–*P*. *pachyrhizi*, 32,520bp–*P*. *meibomiae*). In our case, a comparison of *V*. *nonalfalfae* (26,139bp) with the available mitochondrial genome of the closely related *V*. *dahliae* (27,184bp), showed comparable sizes, a conserved gene order and species specific nucleotide changes, contributing to a 98.15% nucleotide-level identity between mitochondrial genomes.

Our reported *V*. *nonalfalfae* mitochondrial genome is among the smallest published genomes, yielding in total 26,139 bp (max–*S*. *borealis*: 203,051 bp, min*–L*. *muscarium*: 24,499 bp) and has an average GC content of 26.92%, which is quite similar to the GC content of the mitochondrial genome of *V*. *dahliae* (27.32%), although it is still fairly low in terms of the average GC contents of other fungal species considered in this work (29.16%, Table 1). The number of tRNAs seems to be on a par with other members of the *Glomerellales* and *Hypocrea*les groups (Table 1).

Most of the mitochondrial protein-coding genes implicated in oxidative-phosphorylation and ATP synthesis are highly conserved within fungal mitochondrial genomes [10,14,17,21,22]. This also proved to be the case in the *V*. *nonalfalfae* mitochondrial genome, since it harbors the whole complement of 14 "standard" mitochondrial protein-coding genes. Most of the predicted features were placed on the same strand of the mitochondrial genome, with the exception of the long rRNA subunit, which was predicted to be on the opposite strand. Features being mostly encoded on the same strand is very common among Ascomycete mitochondrial genomes [10,14,19,61]. Another interesting feature indicating the taxonomic placement of the *V*. *nonalfalfae* mitochondrial genome is the *rps3* gene, encoded inside an intron, which is reported to be a common feature of the Sordariomycete family of fungal mitochondrial genomes [62]. This gene encodes a ribosomal protein that is a component of the 40S subunit, where it forms part of the domain in which translation is initiated. All of the species analyzed in this study that are members of the Sordariomycetes family contain this intron-encoded *rps3* gene in their mitochondrial genome according to their Genbank entries (*F*. *graminearum*, *F*. *oxysporum*, *F*. *solani*, *L*. *muscarium*, *M*. *chlamydosporia*, *M*. *anisopliae*, *N*. *crassa*, *P*. *anserina*, *R*. *orthosporum*, *S*. *borealis*, *V*. *dahliae*). It seemed at first that *A*. *chrysogenum* lacked an *rps3* gene and *C*. *lindemuthianum* lacked the long rRNA subunit, judging from their Genbank entries, but an in-house analysis revealed both genes in the respective genomes (data not shown).

The gene order for the *V*. *nonalfalfae* mitochondrial genome, starting with *cox2*, is *cox2-atp9-nad6-cox3-atp6-atp8-nad4-nad1-nad3-nad2-cox1-cob-nad5-nad4L*, which is the same gene order as in the *V*. *dahliae* mitochondrial genome. Beyond the conserved gene order, RNA features also show conserved placement, such that tRNAs and rRNAs have almost the same placement on both of these mitochondrial genomes, with the exception of *V*. *dahliae* missing the tRNA-Cys(GCA) and thus having only 25 predicted tRNAs.

These indications of *V*. *nonalfalfae* taxonomic placement were further supported by our sequence-based phylogenetic analysis, which showed how the *V*. *nonalfalfae* mitochondrial genome was positioned in the *Verticillium* spp. lineage of the *Glomerellales* group, with *V*. *dahliae* being the closest relative. Our mitochondrial phylogenetic tree (Fig 4) is concordant with the nuclear genome-based phylogenetic tree, in relation to the Sordariomycetes group [63], even though mitochondria are prone to an accelerated rate of mutation compared to the nuclear genome, together with a higher diversity in gene order and in non-coding regions [13]. An example nuclear-genome based phylogenetic tree [63] shows similar groups of species as those that can be seen in the mitochondrial-based phylogenetic tree, such as the Sordariomycetes comprising the *Verticillium* spp. in the *Glomerellales* group, the *Fusarium* spp. in the *Hypocreales* group and *N*. *crassa* with *P*. *anserina* in the *Sordariales* group. Other groups do not show immediate resemblance with the genome phylogenetic tree but that may be a consequence of low resolution, due to a lack of species of that part of the taxonomy in the phylogenetic analysis.

Another special feature of the *V*. *nonalfalfae* mitochondrial genome is the presence of the potential long non-coding RNA (*orf414*, Fig 1). *orf414* was annotated as a long non-coding RNA because no significant similarity hits were found within the nr database with the BLASTx algorithm. However, it was supported by RNA-Seq data and a further qPCR profiling experiment showed that it was expressed in both Slovenian pathotypes. Running a BLASTn alignment of *orf414* on the fungal genomes of the JGI MycoCosm and NCBI nt databases shows the sequence being unique only to the mitochondrion of the *Verticillium* lineage (*V*. *dahliae*). Aligning the sequence to these mitochondrial genomes showed that its location in *V*. *dahliae* is at the locus between the *cox3* and *nad6* genes, which is the same locus as in *V*. *nonalfalfae*, with a 95% sequence identity. However, an alignment of *orf414* to the *V*. *alfalfae* mitochondrial genome available in the WGS section of the NCBI databases showed only a partial hit, with 40% similarity. Interestingly, in a previous work [64], the same region was used as a tool for differentiating among isolates of *V*. *dahliae*. In that work, the region between the genes *cox3* and *nadh6* (our *orf414*) was found to be highly polymorphic and useful as a marker to separate *V*. *dahliae* isolates into subgroups.

Our comparison of *V*. *nonalfalfae* and *V*. *dahliae* mitochondrial genomes revealed another interesting feature: an additional region (1,221 bp) between the *cox1* gene and a proline-coding tRNA in *V*. *dahliae*. The PCR amplification experiment showed almost all of our *V*. *dahliae* isolates containing that region, together with one isolate of *V*. *nubilum*, which was evident from the amplification of a 1400 bp fragment (S2 Table). In view of these results, this additional region could potentially be used as a discriminator between *V*. *dahliae* and other *Verticillium* species such as *V*. *nonalfalfae*. Current molecular methods of distinguishing among these species rely on sequencing the ITS region of the ribosomal RNA with specific PCR primers [65]. We believe our proposed molecular marker also has the advantage of a multi-copy mitochondrial molecular marker, which might entail faster and more specific pathogen detection directly in soil samples. A molecular marker with similar traits has already been reported for *V*. *dahliae*, which is based on conserved PCR primers in the mitochondrial small rRNA gene region [66].

## Conclusions

Assembly of the *V*. *nonalfalfae* mitochondrial genome resulted in a 26,139 bp DNA molecule with genomic features reminiscent of many other ascomycete fungal mitochondrial genomes, especially to that of *V*. *dahliae*. It harbors a set of 14 oxidative-phosphorylation and ATP synthesis protein-coding genes, small and large rRNA subunits, a ribosomal protein S3 encoded within a type-IA intron and 26 tRNAs, which are common features of Sordariomycete mitochondrial genomes. One interesting additional feature is the presence of a potential long non-coding RNA (*orf414*) in a region that has already been used in a study for differentiating among isolates of *V*. *dahliae* [65]. At this point, the region of *orf414* could only be found in *V*. *dahliae* and *V*. *nonalfalfae*, so we regard it as a unique feature of these 2 species. Using the annotated protein sequences for phylogenetic tree construction, we confirmed this mitochondrial genome to cluster along with that from the closely related *V*. *dahliae* in the *Glomerellales* group of fungi, with a very conserved synteny. This grouping within the *Glomerellales* group is also supported by nuclear genome-data based clustering [63].

The other interesting feature is the additional sequence present in *V*. *dahliae* but absent in *V*. *nonalfalfae*, which might be used as a biomarker to distinguish between these two species.

## Supporting Information

S1 Fig Secondary structures of predicted tRNA molecules. (TIF)

S2 Fig Alignment of *V*. *nonalfalfae* reads to the *V*. *dahliae* mitochondrial genome. (TIF)

S1 File BLAST results of *orf414* analysis. (DOC)

S1 Table V. nonalfalfae codon usage statistics. (DOC)

S2 Table Collection of *Verticill*ium species included in analysis of the mitochondrial length polymorphism. (XLS)
