# Supplementary figures and images for: Complete mitochondrial genome of the Verticillium-wilt causing plant pathogen Verticillium nonalfalfae
Source: PLoS One. 2016 Feb 3;11(2):e0148525. doi: 10.1371/journal.pone.0148525 (PMC4739603; doi:10.1371/journal.pone.0148525)

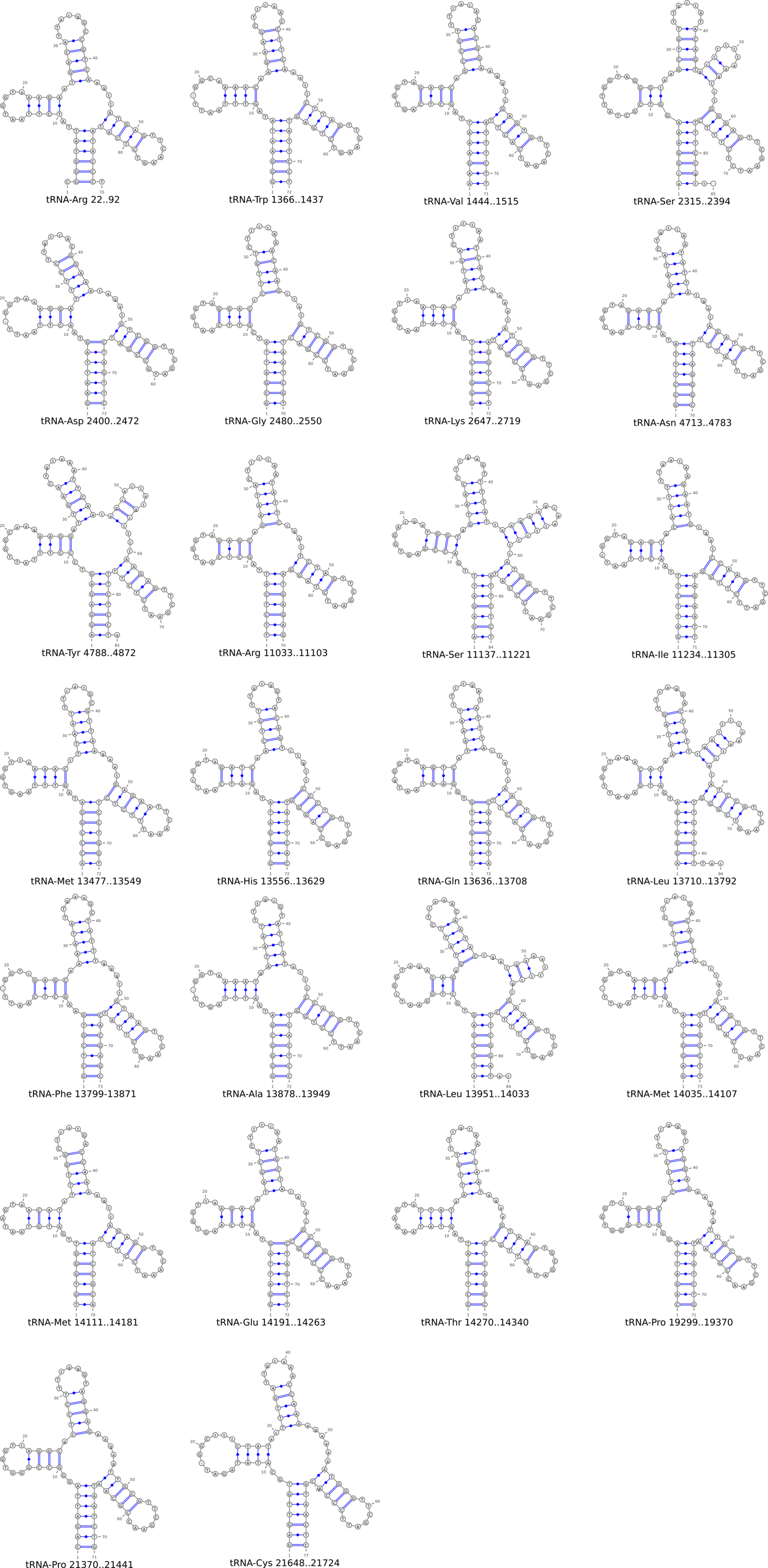

Supplement: S1 Fig — (TIF) [file pone.0148525.s001.tif]

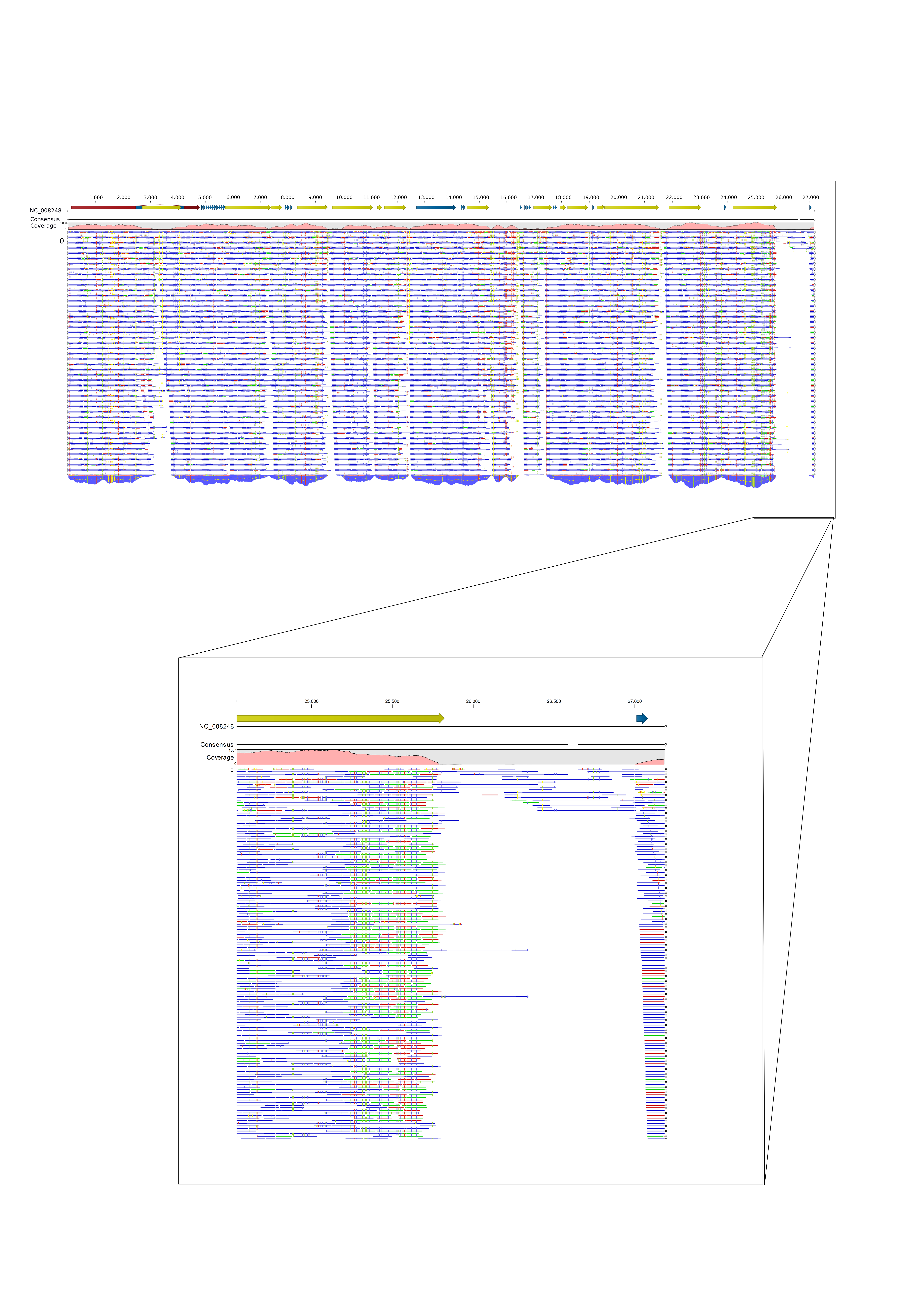

Supplement: S2 Fig — (TIF) [file pone.0148525.s002.tif]
