# Supplementary material for: Complete mitochondrial genome of the Verticillium-wilt causing plant pathogen Verticillium nonalfalfae
Source: PLoS One. 2016 Feb 3;11(2):e0148525. doi: 10.1371/journal.pone.0148525 (PMC4739603; doi:10.1371/journal.pone.0148525)
Supplement: S1 File — (DOC) [file pone.0148525.s003.doc]

Supplementary File 1: BLASTn and BLASTx analysis of *orf414* sequence, e>10^-5^ with >50% of query coverage

BLASTn: Nucleotide collection (nr/nt)

Database: Nucleotide collection (nt)

33,804,645 sequences; 108,060,332,819 total letters

Query= orf414

Length=789

Score E

Sequences producing significant alignments: (Bits) Value

gb|KP994403.1| Verticillium dahliae isolate F624 tRNA-Lys gen... 1276 0.0

gb|KP994402.1| Verticillium dahliae isolate F525 tRNA-Lys gen... 1276 0.0

gb|GU291307.1| Verticillium dahliae isolate V539I cytochrome ... 1276 0.0

gb|GU291306.1| Verticillium dahliae isolate V396I cytochrome ... 1276 0.0

gb|GU291305.1| Verticillium dahliae isolate MP89 cytochrome o... 1276 0.0

gb|GU291304.1| Verticillium dahliae isolate Fca21 cytochrome ... 1276 0.0

gb|KP994401.1| Verticillium dahliae strain V44 tRNA-Lys gene,... 1270 0.0

gb|GU291303.1| Verticillium dahliae isolate V137I cytochrome ... 1270 0.0

gb|DQ351941.1| Verticillium dahliae mitochondrion, complete g... 1267 0.0

gb|CP009079.1| Verticillium dahliae JR2 chromosome 2, complet... 852 0.0

ALIGNMENTS

>gb|KP994403.1| Verticillium dahliae isolate F624 tRNA-Lys gene, partial sequence;

mitochondrial

Length=1060

Score = 1276 bits (1414), Expect = 0.0

Identities = 755/787 (96%), Gaps = 0/787 (0%)

Strand=Plus/Plus

Query 1 CAGAACTCGAACAAGATGATCATGATTCTCATGATGATTCTTTAGCTACTGACCCTGAAG 60

||||||||||||||||||||||||||||||||||||||||||||||||||||||||||||

Sbjct 140 CAGAACTCGAACAAGATGATCATGATTCTCATGATGATTCTTTAGCTACTGACCCTGAAG 199

Query 61 TAGACTCAGGTCTAGACAGCGATGAACAAGTTCAAAGTAGTGGATCCGAGGGAGATGAGA 120

|||||||||||||||| ||||||||||||||||||||||||||||||||||| |||||||

Sbjct 200 TAGACTCAGGTCTAGATAGCGATGAACAAGTTCAAAGTAGTGGATCCGAGGGGGATGAGA 259

Query 121 CTTCAGAAAATGAATCAGAAATTGAAGGTATAAATGAGGTTTCTGATTTTGAAGTTGAAG 180

|||||||||||||||||||||||||||||||||||||||||| ||||||||| |||||||

Sbjct 260 CTTCAGAAAATGAATCAGAAATTGAAGGTATAAATGAGGTTTGTGATTTTGAGGTTGAAG 319

Query 181 AAAGATCAGGTGATAAACTCACAACTGAAAGATTAGCTAATGATCAGACTCATCTTCTTA 240

||||||||||||||||||| | |||||||||||||||||||||||||||||||||||||

Sbjct 320 AAAGATCAGGTGATAAACTTGCTACTGAAAGATTAGCTAATGATCAGACTCATCTTCTTA 379

Query 241 GAGCTTTAAGACATGGAGAACAAGCTTCTATAGATAAAATACAAGAGAGATACCCTGCTT 300

||||||||||||||||||||||||||||||||||||||||||||||||||||||||||||

Sbjct 380 GAGCTTTAAGACATGGAGAACAAGCTTCTATAGATAAAATACAAGAGAGATACCCTGCTT 439

Query 301 TCTTTGATGAAGGTAGTGGAAATTCTTCCATAAAAAAAGGTTTATATCAAGTAAGACATT 360

| ||||||||||| || |||||||||||| ||||||||||||||||||||||||||||||

Sbjct 440 TTTTTGATGAAGGGAGCGGAAATTCTTCCGTAAAAAAAGGTTTATATCAAGTAAGACATT 499

Query 361 ATATAGAAGAAGAATTTGATCTTGAAGAATTAGAAACACTAAAAGAAATAGATAGAGAAG 420

||||||||||||||||||||||||||||||||||||| | |||||||||||||||||||

Sbjct 500 ATATAGAAGAAGAATTTGATCTTGAAGAATTAGAAACCTTGAAAGAAATAGATAGAGAAG 559

Query 421 AAGCTAAACACATAGAAGCAAATAGGTTAATAGAAAAAAATAAACTTGAACTATTTGAAC 480

|||||||| || |||||||||||| |||||||||||||||||||||||||||||| |||

Sbjct 560 AAGCTAAATACCTAGAAGCAAATAAGTTAATAGAAAAAAATAAACTTGAACTATTAGAAT 619

Query 481 CTATAATGAATCCTGAAGAACTTAAACGTAAAAGAGAAGATTCTGAAGAACAATTTGATG 540

|| ||||||||||||||||||||||||||||||||||||||||||||||||||||||||

Sbjct 620 TTACAATGAATCCTGAAGAACTTAAACGTAAAAGAGAAGATTCTGAAGAACAATTTGATG 679

Query 541 AAATTGAAGCTAAAAAAGTAAAAATGAATCCTGAAGAACTTAAACGTAAAAGGGAAGATT 600

|||||||||||||||||||||||||||| |||||||||||||||||||||||||||||||

Sbjct 680 AAATTGAAGCTAAAAAAGTAAAAATGAACCCTGAAGAACTTAAACGTAAAAGGGAAGATT 739

Query 601 TTGAGGAGGTTGAAAATAATCAACCTCCAACTAAAAGAGTAAAAATAAATCATAATAACG 660

|||| | ||||| || ||||||||| |||||||||||||||||||||||||||||| |

Sbjct 740 TTGAAGGAGTTGATGATCATCAACCTCAAACTAAAAGAGTAAAAATAAATCATAATAATG 799

Query 661 ATGATAATAATAACGGACAAAGTGGTATAGGCCCTTGTTCTGGTATTTCATCTGGTACTT 720

|||||||||| ||||||||| |||||||||||||||||||||||||||||||||||||||

Sbjct 800 ATGATAATAACAACGGACAAGGTGGTATAGGCCCTTGTTCTGGTATTTCATCTGGTACTT 859

Query 721 CTTCAGAAGAAACTTCTACTAATGCGCGTAGTATAACTACTTTAATTATTTTATGATTAG 780

||||||||||||||||||||||||||||||||||||||||||||||||||||||||||||

Sbjct 860 CTTCAGAAGAAACTTCTACTAATGCGCGTAGTATAACTACTTTAATTATTTTATGATTAG 919

Query 781 GTAGTAT 787

|||||||

Sbjct 920 GTAGTAT 926

>gb|KP994402.1| Verticillium dahliae isolate F525 tRNA-Lys gene, partial sequence;

mitochondrial

Length=1045

Score = 1276 bits (1414), Expect = 0.0

Identities = 755/787 (96%), Gaps = 0/787 (0%)

Strand=Plus/Plus

Query 1 CAGAACTCGAACAAGATGATCATGATTCTCATGATGATTCTTTAGCTACTGACCCTGAAG 60

||||||||||||||||||||||||||||||||||||||||||||||||||||||||||||

Sbjct 125 CAGAACTCGAACAAGATGATCATGATTCTCATGATGATTCTTTAGCTACTGACCCTGAAG 184

Query 61 TAGACTCAGGTCTAGACAGCGATGAACAAGTTCAAAGTAGTGGATCCGAGGGAGATGAGA 120

|||||||||||||||| ||||||||||||||||||||||||||||||||||| |||||||

Sbjct 185 TAGACTCAGGTCTAGATAGCGATGAACAAGTTCAAAGTAGTGGATCCGAGGGGGATGAGA 244

Query 121 CTTCAGAAAATGAATCAGAAATTGAAGGTATAAATGAGGTTTCTGATTTTGAAGTTGAAG 180

|||||||||||||||||||||||||||||||||||||||||| ||||||||| |||||||

Sbjct 245 CTTCAGAAAATGAATCAGAAATTGAAGGTATAAATGAGGTTTGTGATTTTGAGGTTGAAG 304

Query 181 AAAGATCAGGTGATAAACTCACAACTGAAAGATTAGCTAATGATCAGACTCATCTTCTTA 240

||||||||||||||||||| | |||||||||||||||||||||||||||||||||||||

Sbjct 305 AAAGATCAGGTGATAAACTTGCTACTGAAAGATTAGCTAATGATCAGACTCATCTTCTTA 364

Query 241 GAGCTTTAAGACATGGAGAACAAGCTTCTATAGATAAAATACAAGAGAGATACCCTGCTT 300

||||||||||||||||||||||||||||||||||||||||||||||||||||||||||||

Sbjct 365 GAGCTTTAAGACATGGAGAACAAGCTTCTATAGATAAAATACAAGAGAGATACCCTGCTT 424

Query 301 TCTTTGATGAAGGTAGTGGAAATTCTTCCATAAAAAAAGGTTTATATCAAGTAAGACATT 360

| ||||||||||| || |||||||||||| ||||||||||||||||||||||||||||||

Sbjct 425 TTTTTGATGAAGGGAGCGGAAATTCTTCCGTAAAAAAAGGTTTATATCAAGTAAGACATT 484

Query 361 ATATAGAAGAAGAATTTGATCTTGAAGAATTAGAAACACTAAAAGAAATAGATAGAGAAG 420

||||||||||||||||||||||||||||||||||||| | |||||||||||||||||||

Sbjct 485 ATATAGAAGAAGAATTTGATCTTGAAGAATTAGAAACCTTGAAAGAAATAGATAGAGAAG 544

Query 421 AAGCTAAACACATAGAAGCAAATAGGTTAATAGAAAAAAATAAACTTGAACTATTTGAAC 480

|||||||| || |||||||||||| |||||||||||||||||||||||||||||| |||

Sbjct 545 AAGCTAAATACCTAGAAGCAAATAAGTTAATAGAAAAAAATAAACTTGAACTATTAGAAT 604

Query 481 CTATAATGAATCCTGAAGAACTTAAACGTAAAAGAGAAGATTCTGAAGAACAATTTGATG 540

|| ||||||||||||||||||||||||||||||||||||||||||||||||||||||||

Sbjct 605 TTACAATGAATCCTGAAGAACTTAAACGTAAAAGAGAAGATTCTGAAGAACAATTTGATG 664

Query 541 AAATTGAAGCTAAAAAAGTAAAAATGAATCCTGAAGAACTTAAACGTAAAAGGGAAGATT 600

|||||||||||||||||||||||||||| |||||||||||||||||||||||||||||||

Sbjct 665 AAATTGAAGCTAAAAAAGTAAAAATGAACCCTGAAGAACTTAAACGTAAAAGGGAAGATT 724

Query 601 TTGAGGAGGTTGAAAATAATCAACCTCCAACTAAAAGAGTAAAAATAAATCATAATAACG 660

|||| | ||||| || ||||||||| |||||||||||||||||||||||||||||| |

Sbjct 725 TTGAAGGAGTTGATGATCATCAACCTCAAACTAAAAGAGTAAAAATAAATCATAATAATG 784

Query 661 ATGATAATAATAACGGACAAAGTGGTATAGGCCCTTGTTCTGGTATTTCATCTGGTACTT 720

|||||||||| ||||||||| |||||||||||||||||||||||||||||||||||||||

Sbjct 785 ATGATAATAACAACGGACAAGGTGGTATAGGCCCTTGTTCTGGTATTTCATCTGGTACTT 844

Query 721 CTTCAGAAGAAACTTCTACTAATGCGCGTAGTATAACTACTTTAATTATTTTATGATTAG 780

||||||||||||||||||||||||||||||||||||||||||||||||||||||||||||

Sbjct 845 CTTCAGAAGAAACTTCTACTAATGCGCGTAGTATAACTACTTTAATTATTTTATGATTAG 904

Query 781 GTAGTAT 787

|||||||

Sbjct 905 GTAGTAT 911

>gb|GU291307.1| Verticillium dahliae isolate V539I cytochrome oxidase subunit

III (cox3) gene, partial cds; tRNA-Lys, tRNA-Gly, tRNA-Asp,

and tRNA-Ser genes, complete sequence; and NADH dehydrogenase

subunit 6 (nad6) gene, partial cds; mitochondrial

Length=1728

Score = 1276 bits (1414), Expect = 0.0

Identities = 755/787 (96%), Gaps = 0/787 (0%)

Strand=Plus/Plus

Query 1 CAGAACTCGAACAAGATGATCATGATTCTCATGATGATTCTTTAGCTACTGACCCTGAAG 60

||||||||||||||||||||||||||||||||||||||||||||||||||||||||||||

Sbjct 265 CAGAACTCGAACAAGATGATCATGATTCTCATGATGATTCTTTAGCTACTGACCCTGAAG 324

Query 61 TAGACTCAGGTCTAGACAGCGATGAACAAGTTCAAAGTAGTGGATCCGAGGGAGATGAGA 120

|||||||||||||||| ||||||||||||||||||||||||||||||||||| |||||||

Sbjct 325 TAGACTCAGGTCTAGATAGCGATGAACAAGTTCAAAGTAGTGGATCCGAGGGGGATGAGA 384

Query 121 CTTCAGAAAATGAATCAGAAATTGAAGGTATAAATGAGGTTTCTGATTTTGAAGTTGAAG 180

|||||||||||||||||||||||||||||||||||||||||| ||||||||| |||||||

Sbjct 385 CTTCAGAAAATGAATCAGAAATTGAAGGTATAAATGAGGTTTGTGATTTTGAGGTTGAAG 444

Query 181 AAAGATCAGGTGATAAACTCACAACTGAAAGATTAGCTAATGATCAGACTCATCTTCTTA 240

||||||||||||||||||| | |||||||||||||||||||||||||||||||||||||

Sbjct 445 AAAGATCAGGTGATAAACTTGCTACTGAAAGATTAGCTAATGATCAGACTCATCTTCTTA 504

Query 241 GAGCTTTAAGACATGGAGAACAAGCTTCTATAGATAAAATACAAGAGAGATACCCTGCTT 300

||||||||||||||||||||||||||||||||||||||||||||||||||||||||||||

Sbjct 505 GAGCTTTAAGACATGGAGAACAAGCTTCTATAGATAAAATACAAGAGAGATACCCTGCTT 564

Query 301 TCTTTGATGAAGGTAGTGGAAATTCTTCCATAAAAAAAGGTTTATATCAAGTAAGACATT 360

| ||||||||||| || |||||||||||| ||||||||||||||||||||||||||||||

Sbjct 565 TTTTTGATGAAGGGAGCGGAAATTCTTCCGTAAAAAAAGGTTTATATCAAGTAAGACATT 624

Query 361 ATATAGAAGAAGAATTTGATCTTGAAGAATTAGAAACACTAAAAGAAATAGATAGAGAAG 420

||||||||||||||||||||||||||||||||||||| | |||||||||||||||||||

Sbjct 625 ATATAGAAGAAGAATTTGATCTTGAAGAATTAGAAACCTTGAAAGAAATAGATAGAGAAG 684

Query 421 AAGCTAAACACATAGAAGCAAATAGGTTAATAGAAAAAAATAAACTTGAACTATTTGAAC 480

|||||||| || |||||||||||| |||||||||||||||||||||||||||||| |||

Sbjct 685 AAGCTAAATACCTAGAAGCAAATAAGTTAATAGAAAAAAATAAACTTGAACTATTAGAAT 744

Query 481 CTATAATGAATCCTGAAGAACTTAAACGTAAAAGAGAAGATTCTGAAGAACAATTTGATG 540

|| ||||||||||||||||||||||||||||||||||||||||||||||||||||||||

Sbjct 745 TTACAATGAATCCTGAAGAACTTAAACGTAAAAGAGAAGATTCTGAAGAACAATTTGATG 804

Query 541 AAATTGAAGCTAAAAAAGTAAAAATGAATCCTGAAGAACTTAAACGTAAAAGGGAAGATT 600

|||||||||||||||||||||||||||| |||||||||||||||||||||||||||||||

Sbjct 805 AAATTGAAGCTAAAAAAGTAAAAATGAACCCTGAAGAACTTAAACGTAAAAGGGAAGATT 864

Query 601 TTGAGGAGGTTGAAAATAATCAACCTCCAACTAAAAGAGTAAAAATAAATCATAATAACG 660

|||| | ||||| || ||||||||| |||||||||||||||||||||||||||||| |

Sbjct 865 TTGAAGGAGTTGATGATCATCAACCTCAAACTAAAAGAGTAAAAATAAATCATAATAATG 924

Query 661 ATGATAATAATAACGGACAAAGTGGTATAGGCCCTTGTTCTGGTATTTCATCTGGTACTT 720

|||||||||| ||||||||| |||||||||||||||||||||||||||||||||||||||

Sbjct 925 ATGATAATAACAACGGACAAGGTGGTATAGGCCCTTGTTCTGGTATTTCATCTGGTACTT 984

Query 721 CTTCAGAAGAAACTTCTACTAATGCGCGTAGTATAACTACTTTAATTATTTTATGATTAG 780

||||||||||||||||||||||||||||||||||||||||||||||||||||||||||||

Sbjct 985 CTTCAGAAGAAACTTCTACTAATGCGCGTAGTATAACTACTTTAATTATTTTATGATTAG 1044

Query 781 GTAGTAT 787

|||||||

Sbjct 1045 GTAGTAT 1051

>gb|GU291306.1| Verticillium dahliae isolate V396I cytochrome oxidase subunit

III (cox3) gene, partial cds; tRNA-Lys, tRNA-Gly, tRNA-Asp,

and tRNA-Ser genes, complete sequence; and NADH dehydrogenase

subunit 6 (nad6) gene, partial cds; mitochondrial

Length=1754

Score = 1276 bits (1414), Expect = 0.0

Identities = 755/787 (96%), Gaps = 0/787 (0%)

Strand=Plus/Plus

Query 1 CAGAACTCGAACAAGATGATCATGATTCTCATGATGATTCTTTAGCTACTGACCCTGAAG 60

||||||||||||||||||||||||||||||||||||||||||||||||||||||||||||

Sbjct 276 CAGAACTCGAACAAGATGATCATGATTCTCATGATGATTCTTTAGCTACTGACCCTGAAG 335

Query 61 TAGACTCAGGTCTAGACAGCGATGAACAAGTTCAAAGTAGTGGATCCGAGGGAGATGAGA 120

|||||||||||||||| ||||||||||||||||||||||||||||||||||| |||||||

Sbjct 336 TAGACTCAGGTCTAGATAGCGATGAACAAGTTCAAAGTAGTGGATCCGAGGGGGATGAGA 395

Query 121 CTTCAGAAAATGAATCAGAAATTGAAGGTATAAATGAGGTTTCTGATTTTGAAGTTGAAG 180

|||||||||||||||||||||||||||||||||||||||||| ||||||||| |||||||

Sbjct 396 CTTCAGAAAATGAATCAGAAATTGAAGGTATAAATGAGGTTTGTGATTTTGAGGTTGAAG 455

Query 181 AAAGATCAGGTGATAAACTCACAACTGAAAGATTAGCTAATGATCAGACTCATCTTCTTA 240

||||||||||||||||||| | |||||||||||||||||||||||||||||||||||||

Sbjct 456 AAAGATCAGGTGATAAACTTGCTACTGAAAGATTAGCTAATGATCAGACTCATCTTCTTA 515

Query 241 GAGCTTTAAGACATGGAGAACAAGCTTCTATAGATAAAATACAAGAGAGATACCCTGCTT 300

||||||||||||||||||||||||||||||||||||||||||||||||||||||||||||

Sbjct 516 GAGCTTTAAGACATGGAGAACAAGCTTCTATAGATAAAATACAAGAGAGATACCCTGCTT 575

Query 301 TCTTTGATGAAGGTAGTGGAAATTCTTCCATAAAAAAAGGTTTATATCAAGTAAGACATT 360

| ||||||||||| || |||||||||||| ||||||||||||||||||||||||||||||

Sbjct 576 TTTTTGATGAAGGGAGCGGAAATTCTTCCGTAAAAAAAGGTTTATATCAAGTAAGACATT 635

Query 361 ATATAGAAGAAGAATTTGATCTTGAAGAATTAGAAACACTAAAAGAAATAGATAGAGAAG 420

||||||||||||||||||||||||||||||||||||| | |||||||||||||||||||

Sbjct 636 ATATAGAAGAAGAATTTGATCTTGAAGAATTAGAAACCTTGAAAGAAATAGATAGAGAAG 695

Query 421 AAGCTAAACACATAGAAGCAAATAGGTTAATAGAAAAAAATAAACTTGAACTATTTGAAC 480

|||||||| || |||||||||||| |||||||||||||||||||||||||||||| |||

Sbjct 696 AAGCTAAATACCTAGAAGCAAATAAGTTAATAGAAAAAAATAAACTTGAACTATTAGAAT 755

Query 481 CTATAATGAATCCTGAAGAACTTAAACGTAAAAGAGAAGATTCTGAAGAACAATTTGATG 540

|| ||||||||||||||||||||||||||||||||||||||||||||||||||||||||

Sbjct 756 TTACAATGAATCCTGAAGAACTTAAACGTAAAAGAGAAGATTCTGAAGAACAATTTGATG 815

Query 541 AAATTGAAGCTAAAAAAGTAAAAATGAATCCTGAAGAACTTAAACGTAAAAGGGAAGATT 600

|||||||||||||||||||||||||||| |||||||||||||||||||||||||||||||

Sbjct 816 AAATTGAAGCTAAAAAAGTAAAAATGAACCCTGAAGAACTTAAACGTAAAAGGGAAGATT 875

Query 601 TTGAGGAGGTTGAAAATAATCAACCTCCAACTAAAAGAGTAAAAATAAATCATAATAACG 660

|||| | ||||| || ||||||||| |||||||||||||||||||||||||||||| |

Sbjct 876 TTGAAGGAGTTGATGATCATCAACCTCAAACTAAAAGAGTAAAAATAAATCATAATAATG 935

Query 661 ATGATAATAATAACGGACAAAGTGGTATAGGCCCTTGTTCTGGTATTTCATCTGGTACTT 720

|||||||||| ||||||||| |||||||||||||||||||||||||||||||||||||||

Sbjct 936 ATGATAATAACAACGGACAAGGTGGTATAGGCCCTTGTTCTGGTATTTCATCTGGTACTT 995

Query 721 CTTCAGAAGAAACTTCTACTAATGCGCGTAGTATAACTACTTTAATTATTTTATGATTAG 780

||||||||||||||||||||||||||||||||||||||||||||||||||||||||||||

Sbjct 996 CTTCAGAAGAAACTTCTACTAATGCGCGTAGTATAACTACTTTAATTATTTTATGATTAG 1055

Query 781 GTAGTAT 787

|||||||

Sbjct 1056 GTAGTAT 1062

>gb|GU291305.1| Verticillium dahliae isolate MP89 cytochrome oxidase subunit

III (cox3) gene, partial cds; tRNA-Lys, tRNA-Gly, tRNA-Asp,

and tRNA-Ser genes, complete sequence; and NADH dehydrogenase

subunit 6 (nad6) gene, partial cds; mitochondrial

Length=1662

Score = 1276 bits (1414), Expect = 0.0

Identities = 755/787 (96%), Gaps = 0/787 (0%)

Strand=Plus/Plus

Query 1 CAGAACTCGAACAAGATGATCATGATTCTCATGATGATTCTTTAGCTACTGACCCTGAAG 60

||||||||||||||||||||||||||||||||||||||||||||||||||||||||||||

Sbjct 233 CAGAACTCGAACAAGATGATCATGATTCTCATGATGATTCTTTAGCTACTGACCCTGAAG 292

Query 61 TAGACTCAGGTCTAGACAGCGATGAACAAGTTCAAAGTAGTGGATCCGAGGGAGATGAGA 120

|||||||||||||||| ||||||||||||||||||||||||||||||||||| |||||||

Sbjct 293 TAGACTCAGGTCTAGATAGCGATGAACAAGTTCAAAGTAGTGGATCCGAGGGGGATGAGA 352

Query 121 CTTCAGAAAATGAATCAGAAATTGAAGGTATAAATGAGGTTTCTGATTTTGAAGTTGAAG 180

|||||||||||||||||||||||||||||||||||||||||| ||||||||| |||||||

Sbjct 353 CTTCAGAAAATGAATCAGAAATTGAAGGTATAAATGAGGTTTGTGATTTTGAGGTTGAAG 412

Query 181 AAAGATCAGGTGATAAACTCACAACTGAAAGATTAGCTAATGATCAGACTCATCTTCTTA 240

||||||||||||||||||| | |||||||||||||||||||||||||||||||||||||

Sbjct 413 AAAGATCAGGTGATAAACTTGCTACTGAAAGATTAGCTAATGATCAGACTCATCTTCTTA 472

Query 241 GAGCTTTAAGACATGGAGAACAAGCTTCTATAGATAAAATACAAGAGAGATACCCTGCTT 300

||||||||||||||||||||||||||||||||||||||||||||||||||||||||||||

Sbjct 473 GAGCTTTAAGACATGGAGAACAAGCTTCTATAGATAAAATACAAGAGAGATACCCTGCTT 532

Query 301 TCTTTGATGAAGGTAGTGGAAATTCTTCCATAAAAAAAGGTTTATATCAAGTAAGACATT 360

| ||||||||||| || |||||||||||| ||||||||||||||||||||||||||||||

Sbjct 533 TTTTTGATGAAGGGAGCGGAAATTCTTCCGTAAAAAAAGGTTTATATCAAGTAAGACATT 592

Query 361 ATATAGAAGAAGAATTTGATCTTGAAGAATTAGAAACACTAAAAGAAATAGATAGAGAAG 420

||||||||||||||||||||||||||||||||||||| | |||||||||||||||||||

Sbjct 593 ATATAGAAGAAGAATTTGATCTTGAAGAATTAGAAACCTTGAAAGAAATAGATAGAGAAG 652

Query 421 AAGCTAAACACATAGAAGCAAATAGGTTAATAGAAAAAAATAAACTTGAACTATTTGAAC 480

|||||||| || |||||||||||| |||||||||||||||||||||||||||||| |||

Sbjct 653 AAGCTAAATACCTAGAAGCAAATAAGTTAATAGAAAAAAATAAACTTGAACTATTAGAAT 712

Query 481 CTATAATGAATCCTGAAGAACTTAAACGTAAAAGAGAAGATTCTGAAGAACAATTTGATG 540

|| ||||||||||||||||||||||||||||||||||||||||||||||||||||||||

Sbjct 713 TTACAATGAATCCTGAAGAACTTAAACGTAAAAGAGAAGATTCTGAAGAACAATTTGATG 772

Query 541 AAATTGAAGCTAAAAAAGTAAAAATGAATCCTGAAGAACTTAAACGTAAAAGGGAAGATT 600

|||||||||||||||||||||||||||| |||||||||||||||||||||||||||||||

Sbjct 773 AAATTGAAGCTAAAAAAGTAAAAATGAACCCTGAAGAACTTAAACGTAAAAGGGAAGATT 832

Query 601 TTGAGGAGGTTGAAAATAATCAACCTCCAACTAAAAGAGTAAAAATAAATCATAATAACG 660

|||| | ||||| || ||||||||| |||||||||||||||||||||||||||||| |

Sbjct 833 TTGAAGGAGTTGATGATCATCAACCTCAAACTAAAAGAGTAAAAATAAATCATAATAATG 892

Query 661 ATGATAATAATAACGGACAAAGTGGTATAGGCCCTTGTTCTGGTATTTCATCTGGTACTT 720

|||||||||| ||||||||| |||||||||||||||||||||||||||||||||||||||

Sbjct 893 ATGATAATAACAACGGACAAGGTGGTATAGGCCCTTGTTCTGGTATTTCATCTGGTACTT 952

Query 721 CTTCAGAAGAAACTTCTACTAATGCGCGTAGTATAACTACTTTAATTATTTTATGATTAG 780

||||||||||||||||||||||||||||||||||||||||||||||||||||||||||||

Sbjct 953 CTTCAGAAGAAACTTCTACTAATGCGCGTAGTATAACTACTTTAATTATTTTATGATTAG 1012

Query 781 GTAGTAT 787

|||||||

Sbjct 1013 GTAGTAT 1019

>gb|GU291304.1| Verticillium dahliae isolate Fca21 cytochrome oxidase subunit

III (cox3) gene, partial cds; tRNA-Lys, tRNA-Gly, tRNA-Asp,

and tRNA-Ser genes, complete sequence; and NADH dehydrogenase

subunit 6 (nad6) gene, partial cds; mitochondrial

Length=1644

Score = 1276 bits (1414), Expect = 0.0

Identities = 755/787 (96%), Gaps = 0/787 (0%)

Strand=Plus/Plus

Query 1 CAGAACTCGAACAAGATGATCATGATTCTCATGATGATTCTTTAGCTACTGACCCTGAAG 60

||||||||||||||||||||||||||||||||||||||||||||||||||||||||||||

Sbjct 208 CAGAACTCGAACAAGATGATCATGATTCTCATGATGATTCTTTAGCTACTGACCCTGAAG 267

Query 61 TAGACTCAGGTCTAGACAGCGATGAACAAGTTCAAAGTAGTGGATCCGAGGGAGATGAGA 120

|||||||||||||||| ||||||||||||||||||||||||||||||||||| |||||||

Sbjct 268 TAGACTCAGGTCTAGATAGCGATGAACAAGTTCAAAGTAGTGGATCCGAGGGGGATGAGA 327

Query 121 CTTCAGAAAATGAATCAGAAATTGAAGGTATAAATGAGGTTTCTGATTTTGAAGTTGAAG 180

|||||||||||||||||||||||||||||||||||||||||| ||||||||| |||||||

Sbjct 328 CTTCAGAAAATGAATCAGAAATTGAAGGTATAAATGAGGTTTGTGATTTTGAGGTTGAAG 387

Query 181 AAAGATCAGGTGATAAACTCACAACTGAAAGATTAGCTAATGATCAGACTCATCTTCTTA 240

||||||||||||||||||| | |||||||||||||||||||||||||||||||||||||

Sbjct 388 AAAGATCAGGTGATAAACTTGCTACTGAAAGATTAGCTAATGATCAGACTCATCTTCTTA 447

Query 241 GAGCTTTAAGACATGGAGAACAAGCTTCTATAGATAAAATACAAGAGAGATACCCTGCTT 300

||||||||||||||||||||||||||||||||||||||||||||||||||||||||||||

Sbjct 448 GAGCTTTAAGACATGGAGAACAAGCTTCTATAGATAAAATACAAGAGAGATACCCTGCTT 507

Query 301 TCTTTGATGAAGGTAGTGGAAATTCTTCCATAAAAAAAGGTTTATATCAAGTAAGACATT 360

| ||||||||||| || |||||||||||| ||||||||||||||||||||||||||||||

Sbjct 508 TTTTTGATGAAGGGAGCGGAAATTCTTCCGTAAAAAAAGGTTTATATCAAGTAAGACATT 567

Query 361 ATATAGAAGAAGAATTTGATCTTGAAGAATTAGAAACACTAAAAGAAATAGATAGAGAAG 420

||||||||||||||||||||||||||||||||||||| | |||||||||||||||||||

Sbjct 568 ATATAGAAGAAGAATTTGATCTTGAAGAATTAGAAACCTTGAAAGAAATAGATAGAGAAG 627

Query 421 AAGCTAAACACATAGAAGCAAATAGGTTAATAGAAAAAAATAAACTTGAACTATTTGAAC 480

|||||||| || |||||||||||| |||||||||||||||||||||||||||||| |||

Sbjct 628 AAGCTAAATACCTAGAAGCAAATAAGTTAATAGAAAAAAATAAACTTGAACTATTAGAAT 687

Query 481 CTATAATGAATCCTGAAGAACTTAAACGTAAAAGAGAAGATTCTGAAGAACAATTTGATG 540

|| ||||||||||||||||||||||||||||||||||||||||||||||||||||||||

Sbjct 688 TTACAATGAATCCTGAAGAACTTAAACGTAAAAGAGAAGATTCTGAAGAACAATTTGATG 747

Query 541 AAATTGAAGCTAAAAAAGTAAAAATGAATCCTGAAGAACTTAAACGTAAAAGGGAAGATT 600

|||||||||||||||||||||||||||| |||||||||||||||||||||||||||||||

Sbjct 748 AAATTGAAGCTAAAAAAGTAAAAATGAACCCTGAAGAACTTAAACGTAAAAGGGAAGATT 807

Query 601 TTGAGGAGGTTGAAAATAATCAACCTCCAACTAAAAGAGTAAAAATAAATCATAATAACG 660

|||| | ||||| || ||||||||| |||||||||||||||||||||||||||||| |

Sbjct 808 TTGAAGGAGTTGATGATCATCAACCTCAAACTAAAAGAGTAAAAATAAATCATAATAATG 867

Query 661 ATGATAATAATAACGGACAAAGTGGTATAGGCCCTTGTTCTGGTATTTCATCTGGTACTT 720

|||||||||| ||||||||| |||||||||||||||||||||||||||||||||||||||

Sbjct 868 ATGATAATAACAACGGACAAGGTGGTATAGGCCCTTGTTCTGGTATTTCATCTGGTACTT 927

Query 721 CTTCAGAAGAAACTTCTACTAATGCGCGTAGTATAACTACTTTAATTATTTTATGATTAG 780

||||||||||||||||||||||||||||||||||||||||||||||||||||||||||||

Sbjct 928 CTTCAGAAGAAACTTCTACTAATGCGCGTAGTATAACTACTTTAATTATTTTATGATTAG 987

Query 781 GTAGTAT 787

|||||||

Sbjct 988 GTAGTAT 994

>gb|KP994401.1| Verticillium dahliae strain V44 tRNA-Lys gene, partial sequence;

mitochondrial

Length=1060

Score = 1270 bits (1408), Expect = 0.0

Identities = 754/787 (96%), Gaps = 0/787 (0%)

Strand=Plus/Plus

Query 1 CAGAACTCGAACAAGATGATCATGATTCTCATGATGATTCTTTAGCTACTGACCCTGAAG 60

||||||||||||||||||||||||||||||||||||||||||||||||||||||||||||

Sbjct 140 CAGAACTCGAACAAGATGATCATGATTCTCATGATGATTCTTTAGCTACTGACCCTGAAG 199

Query 61 TAGACTCAGGTCTAGACAGCGATGAACAAGTTCAAAGTAGTGGATCCGAGGGAGATGAGA 120

|||||||||||||||| ||||||||||||||||||||||||||||||||||| |||||||

Sbjct 200 TAGACTCAGGTCTAGATAGCGATGAACAAGTTCAAAGTAGTGGATCCGAGGGGGATGAGA 259

Query 121 CTTCAGAAAATGAATCAGAAATTGAAGGTATAAATGAGGTTTCTGATTTTGAAGTTGAAG 180

|||||||||||||||||||||||||||||||||||||||||| ||||||||| |||||||

Sbjct 260 CTTCAGAAAATGAATCAGAAATTGAAGGTATAAATGAGGTTTGTGATTTTGAGGTTGAAG 319

Query 181 AAAGATCAGGTGATAAACTCACAACTGAAAGATTAGCTAATGATCAGACTCATCTTCTTA 240

||||||||||||||||||| | |||||||||||||||||||||||||||||||||||||

Sbjct 320 AAAGATCAGGTGATAAACTTGCTACTGAAAGATTAGCTAATGATCAGACTCATCTTCTTA 379

Query 241 GAGCTTTAAGACATGGAGAACAAGCTTCTATAGATAAAATACAAGAGAGATACCCTGCTT 300

||||||||||||||||||||||||||||||||||||||||||||||||||||||||||||

Sbjct 380 GAGCTTTAAGACATGGAGAACAAGCTTCTATAGATAAAATACAAGAGAGATACCCTGCTT 439

Query 301 TCTTTGATGAAGGTAGTGGAAATTCTTCCATAAAAAAAGGTTTATATCAAGTAAGACATT 360

| ||||||||||| || |||||||||||| ||||||||||||||||||||||||||||||

Sbjct 440 TTTTTGATGAAGGGAGCGGAAATTCTTCCGTAAAAAAAGGTTTATATCAAGTAAGACATT 499

Query 361 ATATAGAAGAAGAATTTGATCTTGAAGAATTAGAAACACTAAAAGAAATAGATAGAGAAG 420

||||||||||||||||||||||||||||||||||||| | |||||||||||||||||||

Sbjct 500 ATATAGAAGAAGAATTTGATCTTGAAGAATTAGAAACCTTGAAAGAAATAGATAGAGAAG 559

Query 421 AAGCTAAACACATAGAAGCAAATAGGTTAATAGAAAAAAATAAACTTGAACTATTTGAAC 480

|||||||| || |||||||||||| |||||||||||||||||||||||||||||| |||

Sbjct 560 AAGCTAAATACCTAGAAGCAAATAAGTTAATAGAAAAAAATAAACTTGAACTATTAGAAT 619

Query 481 CTATAATGAATCCTGAAGAACTTAAACGTAAAAGAGAAGATTCTGAAGAACAATTTGATG 540

|| ||||||||||||||||||||||||||||||||||||||||||||||||||||||||

Sbjct 620 TTACAATGAATCCTGAAGAACTTAAACGTAAAAGAGAAGATTCTGAAGAACAATTTGATG 679

Query 541 AAATTGAAGCTAAAAAAGTAAAAATGAATCCTGAAGAACTTAAACGTAAAAGGGAAGATT 600

|||||||||||||||||||||||||||| |||||||||||||||||||||||||||||||

Sbjct 680 AAATTGAAGCTAAAAAAGTAAAAATGAACCCTGAAGAACTTAAACGTAAAAGGGAAGATT 739

Query 601 TTGAGGAGGTTGAAAATAATCAACCTCCAACTAAAAGAGTAAAAATAAATCATAATAACG 660

|||| | ||||| || ||||| ||| |||||||||||||||||||||||||||||| |

Sbjct 740 TTGAAGGAGTTGATGATCATCAATCTCAAACTAAAAGAGTAAAAATAAATCATAATAATG 799

Query 661 ATGATAATAATAACGGACAAAGTGGTATAGGCCCTTGTTCTGGTATTTCATCTGGTACTT 720

|||||||||| ||||||||| |||||||||||||||||||||||||||||||||||||||

Sbjct 800 ATGATAATAACAACGGACAAGGTGGTATAGGCCCTTGTTCTGGTATTTCATCTGGTACTT 859

Query 721 CTTCAGAAGAAACTTCTACTAATGCGCGTAGTATAACTACTTTAATTATTTTATGATTAG 780

||||||||||||||||||||||||||||||||||||||||||||||||||||||||||||

Sbjct 860 CTTCAGAAGAAACTTCTACTAATGCGCGTAGTATAACTACTTTAATTATTTTATGATTAG 919

Query 781 GTAGTAT 787

|||||||

Sbjct 920 GTAGTAT 926

>gb|GU291303.1| Verticillium dahliae isolate V137I cytochrome oxidase subunit

III (cox3) gene, partial cds; tRNA-Lys, tRNA-Gly, tRNA-Asp,

and tRNA-Ser genes, complete sequence; and NADH dehydrogenase

subunit 6 (nad6) gene, partial cds; mitochondrial

Length=1715

Score = 1270 bits (1408), Expect = 0.0

Identities = 754/787 (96%), Gaps = 0/787 (0%)

Strand=Plus/Plus

Query 1 CAGAACTCGAACAAGATGATCATGATTCTCATGATGATTCTTTAGCTACTGACCCTGAAG 60

||||||||||||||||||||||||||||||||||||||||||||||||||||||||||||

Sbjct 267 CAGAACTCGAACAAGATGATCATGATTCTCATGATGATTCTTTAGCTACTGACCCTGAAG 326

Query 61 TAGACTCAGGTCTAGACAGCGATGAACAAGTTCAAAGTAGTGGATCCGAGGGAGATGAGA 120

|||||||||||||||| ||||||||||||||||||||||||||||||||||| |||||||

Sbjct 327 TAGACTCAGGTCTAGATAGCGATGAACAAGTTCAAAGTAGTGGATCCGAGGGGGATGAGA 386

Query 121 CTTCAGAAAATGAATCAGAAATTGAAGGTATAAATGAGGTTTCTGATTTTGAAGTTGAAG 180

|||||||||||||||||||||||||||||||||||||||||| ||||||||| |||||||

Sbjct 387 CTTCAGAAAATGAATCAGAAATTGAAGGTATAAATGAGGTTTGTGATTTTGAGGTTGAAG 446

Query 181 AAAGATCAGGTGATAAACTCACAACTGAAAGATTAGCTAATGATCAGACTCATCTTCTTA 240

||||||||||||||||||| | |||||||||||||||||||||||||||||||||||||

Sbjct 447 AAAGATCAGGTGATAAACTTGCTACTGAAAGATTAGCTAATGATCAGACTCATCTTCTTA 506

Query 241 GAGCTTTAAGACATGGAGAACAAGCTTCTATAGATAAAATACAAGAGAGATACCCTGCTT 300

||||||||||||||||||||||||||||||||||||||||||||||||||||||||||||

Sbjct 507 GAGCTTTAAGACATGGAGAACAAGCTTCTATAGATAAAATACAAGAGAGATACCCTGCTT 566

Query 301 TCTTTGATGAAGGTAGTGGAAATTCTTCCATAAAAAAAGGTTTATATCAAGTAAGACATT 360

| ||||||||||| || |||||||||||| ||||||||||||||||||||||||||||||

Sbjct 567 TTTTTGATGAAGGGAGCGGAAATTCTTCCGTAAAAAAAGGTTTATATCAAGTAAGACATT 626

Query 361 ATATAGAAGAAGAATTTGATCTTGAAGAATTAGAAACACTAAAAGAAATAGATAGAGAAG 420

||||||||||||||||||||||||||||||||||||| | |||||||||||||||||||

Sbjct 627 ATATAGAAGAAGAATTTGATCTTGAAGAATTAGAAACCTTGAAAGAAATAGATAGAGAAG 686

Query 421 AAGCTAAACACATAGAAGCAAATAGGTTAATAGAAAAAAATAAACTTGAACTATTTGAAC 480

|||||||| || |||||||||||| |||||||||||||||||||||||||||||| |||

Sbjct 687 AAGCTAAATACCTAGAAGCAAATAAGTTAATAGAAAAAAATAAACTTGAACTATTAGAAT 746

Query 481 CTATAATGAATCCTGAAGAACTTAAACGTAAAAGAGAAGATTCTGAAGAACAATTTGATG 540

|| ||||||||||||||||||||||||||||||||||||||||||||||||||||||||

Sbjct 747 TTACAATGAATCCTGAAGAACTTAAACGTAAAAGAGAAGATTCTGAAGAACAATTTGATG 806

Query 541 AAATTGAAGCTAAAAAAGTAAAAATGAATCCTGAAGAACTTAAACGTAAAAGGGAAGATT 600

|||||||||||||||||||||||||||| |||||||||||||||||||||||||||||||

Sbjct 807 AAATTGAAGCTAAAAAAGTAAAAATGAACCCTGAAGAACTTAAACGTAAAAGGGAAGATT 866

Query 601 TTGAGGAGGTTGAAAATAATCAACCTCCAACTAAAAGAGTAAAAATAAATCATAATAACG 660

|||| | ||||| || ||||| ||| |||||||||||||||||||||||||||||| |

Sbjct 867 TTGAAGGAGTTGATGATCATCAATCTCAAACTAAAAGAGTAAAAATAAATCATAATAATG 926

Query 661 ATGATAATAATAACGGACAAAGTGGTATAGGCCCTTGTTCTGGTATTTCATCTGGTACTT 720

|||||||||| ||||||||| |||||||||||||||||||||||||||||||||||||||

Sbjct 927 ATGATAATAACAACGGACAAGGTGGTATAGGCCCTTGTTCTGGTATTTCATCTGGTACTT 986

Query 721 CTTCAGAAGAAACTTCTACTAATGCGCGTAGTATAACTACTTTAATTATTTTATGATTAG 780

||||||||||||||||||||||||||||||||||||||||||||||||||||||||||||

Sbjct 987 CTTCAGAAGAAACTTCTACTAATGCGCGTAGTATAACTACTTTAATTATTTTATGATTAG 1046

Query 781 GTAGTAT 787

|||||||

Sbjct 1047 GTAGTAT 1053

>gb|DQ351941.1| Verticillium dahliae mitochondrion, complete genome

Length=27184

Score = 1267 bits (1404), Expect = 0.0

Identities = 753/787 (96%), Gaps = 0/787 (0%)

Strand=Plus/Plus

Query 1 CAGAACTCGAACAAGATGATCATGATTCTCATGATGATTCTTTAGCTACTGACCCTGAAG 60

||||||||||||||||||||||||||||||||||||||||||||||||||||||||||||

Sbjct 15557 CAGAACTCGAACAAGATGATCATGATTCTCATGATGATTCTTTAGCTACTGACCCTGAAG 15616

Query 61 TAGACTCAGGTCTAGACAGCGATGAACAAGTTCAAAGTAGTGGATCCGAGGGAGATGAGA 120

|||||||||||||||| ||||||||||||||||||||||||||||||||||| |||||||

Sbjct 15617 TAGACTCAGGTCTAGATAGCGATGAACAAGTTCAAAGTAGTGGATCCGAGGGGGATGAGA 15676

Query 121 CTTCAGAAAATGAATCAGAAATTGAAGGTATAAATGAGGTTTCTGATTTTGAAGTTGAAG 180

|||||||||||||||||||||||||||||||||||||||||| ||||||||| |||||||

Sbjct 15677 CTTCAGAAAATGAATCAGAAATTGAAGGTATAAATGAGGTTTGTGATTTTGAGGTTGAAG 15736

Query 181 AAAGATCAGGTGATAAACTCACAACTGAAAGATTAGCTAATGATCAGACTCATCTTCTTA 240

||||||||||||||||||| | |||||||||||||||||||||||||||||||||||||

Sbjct 15737 AAAGATCAGGTGATAAACTTGCTACTGAAAGATTAGCTAATGATCAGACTCATCTTCTTA 15796

Query 241 GAGCTTTAAGACATGGAGAACAAGCTTCTATAGATAAAATACAAGAGAGATACCCTGCTT 300

||||||||||||||||||||||||||||||||||||||||||||||||||||||||||||

Sbjct 15797 GAGCTTTAAGACATGGAGAACAAGCTTCTATAGATAAAATACAAGAGAGATACCCTGCTT 15856

Query 301 TCTTTGATGAAGGTAGTGGAAATTCTTCCATAAAAAAAGGTTTATATCAAGTAAGACATT 360

| ||||||||||| || |||||||||||| |||||||||||||||||||||||||| |||

Sbjct 15857 TTTTTGATGAAGGGAGCGGAAATTCTTCCGTAAAAAAAGGTTTATATCAAGTAAGAAATT 15916

Query 361 ATATAGAAGAAGAATTTGATCTTGAAGAATTAGAAACACTAAAAGAAATAGATAGAGAAG 420

||||||||||||||||||||||||||||||||||||| | |||||||||||||||||||

Sbjct 15917 ATATAGAAGAAGAATTTGATCTTGAAGAATTAGAAACCTTGAAAGAAATAGATAGAGAAG 15976

Query 421 AAGCTAAACACATAGAAGCAAATAGGTTAATAGAAAAAAATAAACTTGAACTATTTGAAC 480

|||||||| || |||||||||||| || ||||||||||||||||||||||||||| |||

Sbjct 15977 AAGCTAAATACCTAGAAGCAAATAAGTGAATAGAAAAAAATAAACTTGAACTATTAGAAT 16036

Query 481 CTATAATGAATCCTGAAGAACTTAAACGTAAAAGAGAAGATTCTGAAGAACAATTTGATG 540

|| ||||||||||||||||||||||||||||||||||||||||||||||||||||||||

Sbjct 16037 TTACAATGAATCCTGAAGAACTTAAACGTAAAAGAGAAGATTCTGAAGAACAATTTGATG 16096

Query 541 AAATTGAAGCTAAAAAAGTAAAAATGAATCCTGAAGAACTTAAACGTAAAAGGGAAGATT 600

|||||||||||||||||||||||||||| |||||||||||||||||||||||||||||||

Sbjct 16097 AAATTGAAGCTAAAAAAGTAAAAATGAACCCTGAAGAACTTAAACGTAAAAGGGAAGATT 16156

Query 601 TTGAGGAGGTTGAAAATAATCAACCTCCAACTAAAAGAGTAAAAATAAATCATAATAACG 660

|||| | ||||| || ||||||||| |||||||||||||||||||||||||||||| |

Sbjct 16157 TTGAAGGAGTTGATGATCATCAACCTCAAACTAAAAGAGTAAAAATAAATCATAATAATG 16216

Query 661 ATGATAATAATAACGGACAAAGTGGTATAGGCCCTTGTTCTGGTATTTCATCTGGTACTT 720

|||||||||| ||||||||| |||||||||||||||||||||||||||||||||||||||

Sbjct 16217 ATGATAATAACAACGGACAAGGTGGTATAGGCCCTTGTTCTGGTATTTCATCTGGTACTT 16276

Query 721 CTTCAGAAGAAACTTCTACTAATGCGCGTAGTATAACTACTTTAATTATTTTATGATTAG 780

||||||||||||||||||||||||||||||||||||||||||||||||||||||||||||

Sbjct 16277 CTTCAGAAGAAACTTCTACTAATGCGCGTAGTATAACTACTTTAATTATTTTATGATTAG 16336

Query 781 GTAGTAT 787

|||||||

Sbjct 16337 GTAGTAT 16343

>gb|CP009079.1| Verticillium dahliae JR2 chromosome 2, complete sequence

Length=4277765

Score = 852 bits (944), Expect = 0.0

Identities = 533/573 (93%), Gaps = 3/573 (1%)

Strand=Plus/Plus

Query 1 CAGAACTCGAACAAGATGATCATGATTCTCATGATGATTCTTTAGCTACTGACCCTGAAG 60

||||||||||||||||||||||||||||||||||||||||||||||||||||||||||||

Sbjct 3466907 CAGAACTCGAACAAGATGATCATGATTCTCATGATGATTCTTTAGCTACTGACCCTGAAG 3466966

Query 61 TAGACTCAGGTCTAGACAGCGATGAACAAGTTCAAAGTAGTGGATCCGAGGGAGATGAGA 120

|||||||||||||||| ||||||||||||||||||||||||||||||||||| |||||||

Sbjct 3466967 TAGACTCAGGTCTAGATAGCGATGAACAAGTTCAAAGTAGTGGATCCGAGGGGGATGAGA 3467026

Query 121 CTTCAGAAAATGAATCAGAAATTGAAGGTATAAATGAGGTTTCTGATTTTGAAGTTGAAG 180

|||||||||||||||||||||||||||||||||||||||||| ||||||||| |||||||

Sbjct 3467027 CTTCAGAAAATGAATCAGAAATTGAAGGTATAAATGAGGTTTGTGATTTTGAGGTTGAAG 3467086

Query 181 AAAGATCAGGTGATAAACTCACAACTGAAAGATTAGCTAATGATCAGACTCATCTTCTTA 240

||||||||||||||||||| | |||||||||||||||||||||||||||||||||||||

Sbjct 3467087 AAAGATCAGGTGATAAACTTGCTACTGAAAGATTAGCTAATGATCAGACTCATCTTCTTA 3467146

Query 241 GAGCTTTAAGACATGGAGAACAAGCTTCTATAGATAAAATACAAGAGAGATACCCTGCTT 300

||||||||||||||||||||||||||||||||||||||||||||||||||||||||||||

Sbjct 3467147 GAGCTTTAAGACATGGAGAACAAGCTTCTATAGATAAAATACAAGAGAGATACCCTGCTT 3467206

Query 301 TCTTTGATGAAGGTAGTGGAAATTCTTCCATAAAAAAAGGTTTATATCAAGTAAGACATT 360

| ||||||||||| || |||||||||||| ||||||||||||||||||||||||||||||

Sbjct 3467207 TTTTTGATGAAGGGAGCGGAAATTCTTCCGTAAAAAAAGGTTTATATCAAGTAAGACATT 3467266

Query 361 ATATAGAAGAAGAATTTGATCTTGAAGAATTAGAAACACTAAAAGAAATAGATAGAGAAG 420

||||||||||||||||||||||||||||||||||||| | |||||||||||||||||||

Sbjct 3467267 ATATAGAAGAAGAATTTGATCTTGAAGAATTAGAAACCTTGAAAGAAATAGATAGAGAAG 3467326

Query 421 AAGCTAAACACATAGAAGCAAATAGGTTAATAGAAAAAAATAAACTTGAACTATTTGAAC 480

|||||||| || |||||||||||| |||||||||||||||||||||||||||||| |||

Sbjct 3467327 AAGCTAAATACCTAGAAGCAAATAAGTTAATAGAAAAAAATAAACTTGAACTATTAGAAT 3467386

Query 481 CTATAATGAATCCTGAAGAACTTAAACGTAAAAGAGAAGATTCTGAAGAA---CAATTTG 537

|| ||||||||| |||||||||||||||||||| ||||||| ||||| | | |

Sbjct 3467387 TTACAATGAATCCAGAAGAACTTAAACGTAAAAGGGAAGATTTTGAAGGAGTTGATAATC 3467446

Query 538 ATGAAATTGAAGCTAAAAAAGTAAAAATGAATC 570

|| || | || |||||| ||||||||| ||||

Sbjct 3467447 ATCAACCTCAAACTAAAAGAGTAAAAATAAATC 3467479

Score = 174 bits (192), Expect = 3e-39

Identities = 113/124 (91%), Gaps = 0/124 (0%)

Strand=Plus/Plus

Query 563 AATGAATCCTGAAGAACTTAAACGTAAAAGGGAAGATTTTGAGGAGGTTGAAAATAATCA 622

||||||||| |||||||||||||||||||||||||||||||| | ||||| ||| ||||

Sbjct 3467391 AATGAATCCAGAAGAACTTAAACGTAAAAGGGAAGATTTTGAAGGAGTTGATAATCATCA 3467450

Query 623 ACCTCCAACTAAAAGAGTAAAAATAAATCATAATAACGATGATAATAATAACGGACAAAG 682

||||| |||||||||||||||||||||||||||||| ||| ||||||| ||||||||| |

Sbjct 3467451 ACCTCAAACTAAAAGAGTAAAAATAAATCATAATAATGATAATAATAACAACGGACAAGG 3467510

Query 683 TGGT 686

||||

Sbjct 3467511 TGGT 3467514

BLASTn: Reference RNA sequences (refseq_rna)

No significant similarity found.

BLASTn: Reference genomic sequences (refseq_genomic)

Database: NCBI Genomic Reference Sequences

16,767,017 sequences; 729,514,999,410 total letters

Query= orf414

Length=789

Score E

Sequences producing significant alignments: (Bits) Value

ref|NW_009276970.1| Verticillium dahliae VdLs.17 supercont1.53 m... 1282 0.0

ref|NW_009276969.1| Verticillium dahliae VdLs.17 supercont1.54 m... 1275 0.0

ref|NC_008248.1| Verticillium dahliae mitochondrion, complete ge... 1271 0.0

ref|NW_009276924.1| Verticillium dahliae VdLs.17 supercont1.21 g... 246 3e-060

ref|NW_003315033.1| Verticillium albo-atrum VaMs.102 supercont1.... 80.5 3e-010

>ref|NW_009276970.1| Verticillium dahliae VdLs.17 supercont1.53 mitochondrial scaffold,

whole genome shotgun sequence

Length=42389

Score = 1282 bits (694), Expect = 0.0

Identities = 757/788 (96%), Gaps = 2/788 (0%)

Strand=Plus/Minus

Query 1 CAGAACTCGAACAAGATGATCATGATTCTCATGATGATTCTTTAGCTACTGACCCTGAAG 60

||||||||||||||||||||||||||||||||||||||||||||||||||||||||||||

Sbjct 6084 CAGAACTCGAACAAGATGATCATGATTCTCATGATGATTCTTTAGCTACTGACCCTGAAG 6025

Query 61 TAGACTCAGGTCTAGACAGCGATGAACAAGTTCAAAGTAGTGGATCCGAGGGAGATGAGA 120

|||||||||||||||| ||||||||||||||||||||||||||||||||||| |||||||

Sbjct 6024 TAGACTCAGGTCTAGATAGCGATGAACAAGTTCAAAGTAGTGGATCCGAGGGGGATGAGA 5965

Query 121 CTTCAGAAAATGAATCAGAAATTGAAGGTATAAATGAGGTTTCTGATTTTGAAGTTGAAG 180

|||||||||||||||||||||||||||||||||||||||||| ||||||||| |||||||

Sbjct 5964 CTTCAGAAAATGAATCAGAAATTGAAGGTATAAATGAGGTTTGTGATTTTGAGGTTGAAG 5905

Query 181 AAAGATCAGGTGATAAACTCACAACTGAAAGATTAGCTAATGATCAGACTCATCTTCTTA 240

||||||||||||||||||| | |||||||||||||||||||||||||||||||||||||

Sbjct 5904 AAAGATCAGGTGATAAACTTGCTACTGAAAGATTAGCTAATGATCAGACTCATCTTCTTA 5845

Query 241 GAGCTTTAAGACATGGAGAACAAGCTTCTATAGATAAAATACAAGAGAGATACCCTGCTT 300

||||||||||||||||||||||||||||||||||||||||||||||||||||||||||||

Sbjct 5844 GAGCTTTAAGACATGGAGAACAAGCTTCTATAGATAAAATACAAGAGAGATACCCTGCTT 5785

Query 301 TCTTTGATGAAGGTAGTGGAAATTCTTCCATAAAAAAAGGTTTATATCAAGTAAGACATT 360

| ||||||||||| || |||||||||||| ||||||||||||||||||||||||||||||

Sbjct 5784 TTTTTGATGAAGGGAGCGGAAATTCTTCCGTAAAAAAAGGTTTATATCAAGTAAGACATT 5725

Query 361 ATATAGAAGAAGAATTTGATCTTGAAGAATTAGAAACACTAAAAGAAATAGATAGAGAAG 420

||||||||||||||||||||||||||||||||||||| | |||||||||||||||||||

Sbjct 5724 ATATAGAAGAAGAATTTGATCTTGAAGAATTAGAAACCTTGAAAGAAATAGATAGAGAAG 5665

Query 421 AAGCTAAACACATAGAAGCAAATAGGTTAATAGAAAAAAATAAACTTGAACTATTTGAAC 480

|||||||| || |||||||||||| |||||||||||||||||||||||||||||| |||

Sbjct 5664 AAGCTAAATACCTAGAAGCAAATAAGTTAATAGAAAAAAATAAACTTGAACTATTAGAAT 5605

Query 481 CTATAATGAATCCTGAAGAACTTAAACGTAAAAGAGAAGATTCTGAAGAACAATTTGATG 540

|| ||||||||||||||||||||||||||||||||||||||||||||||||||||||||

Sbjct 5604 TTACAATGAATCCTGAAGAACTTAAACGTAAAAGAGAAGATTCTGAAGAACAATTTGATG 5545

Query 541 AAATTGAAGCTAAAAAAGTAAAAATGAATCCTGAAGAACTTAAACGTAAAAGGGAAGATT 600

|||||||||||||||||||||||||||| |||||||||||||||||||||||||||||||

Sbjct 5544 AAATTGAAGCTAAAAAAGTAAAAATGAACCCTGAAGAACTTAAACGTAAAAGGGAAGATT 5485

Query 601 TTGA-GGAGGTTGAAAATAATCAACCTCCAACTAAAAGAGTAAAAATAAATCATAATAAC 659

|||| ||| ||||| || ||||||||| ||||||||||||||||||||||||||||||

Sbjct 5484 TTGAAGGA-GTTGATGATCATCAACCTCAAACTAAAAGAGTAAAAATAAATCATAATAAT 5426

Query 660 GATGATAATAATAACGGACAAAGTGGTATAGGCCCTTGTTCTGGTATTTCATCTGGTACT 719

||||||||||| ||||||||| ||||||||||||||||||||||||||||||||||||||

Sbjct 5425 GATGATAATAACAACGGACAAGGTGGTATAGGCCCTTGTTCTGGTATTTCATCTGGTACT 5366

Query 720 TCTTCAGAAGAAACTTCTACTAATGCGCGTAGTATAACTACTTTAATTATTTTATGATTA 779

||||||||||||||||||||||||||||||||||||||||||||||||||||||||||||

Sbjct 5365 TCTTCAGAAGAAACTTCTACTAATGCGCGTAGTATAACTACTTTAATTATTTTATGATTA 5306

Query 780 GGTAGTAT 787

||||||||

Sbjct 5305 GGTAGTAT 5298

Score = 1275 bits (690), Expect = 0.0

Identities = 756/788 (96%), Gaps = 3/788 (0%)

Strand=Plus/Minus

Query 1 CAGAACTCGAACAAGATGATCATGATTCTCATGATGATTCTTTAGCTACTGACCCTGAAG 60

||||||||||||||||||||||||||||||||||||||||||||||||||||||||||||

Sbjct 33051 CAGAACTCGAACAAGATGATCATGATTCTCATGATGATTCTTTAGCTACTGACCCTGAAG 32992

Query 61 TAGACTCAGGTCTAGACAGCGATGAACAAGTTCAAAGTAGTGGATCCGAGGGAGATGAGA 120

|||||||||||||||| ||||||||||||||||||||||||||||||||||| |||||||

Sbjct 32991 TAGACTCAGGTCTAGATAGCGATGAACAAGTTCAAAGTAGTGGATCCGAGGGGGATGAGA 32932

Query 121 CTTCAGAAAATGAATCAGAAATTGAAGGTATAAATGAGGTTTCTGATTTTGAAGTTGAAG 180

|||||||||||||||||||||||||||||||||||||||||| ||||||||| |||||||

Sbjct 32931 CTTCAGAAAATGAATCAGAAATTGAAGGTATAAATGAGGTTTGTGATTTTGAGGTTGAAG 32872

Query 181 AAAGATCAGGTGATAAACTCACAACTGAAAGATTAGCTAATGATCAGACTCATCTTCTTA 240

||||||||||||||||||| | |||||||||||||||||||||||||||||||||||||

Sbjct 32871 AAAGATCAGGTGATAAACTTGCTACTGAAAGATTAGCTAATGATCAGACTCATCTTCTTA 32812

Query 241 GAGCTTTAAGACATGGAGAACAAGCTTCTATAGATAAAATACAAGAGAGATACCCTGCTT 300

||||||||||||||||||||||||||||||||||||||||||||||||||||||||||||

Sbjct 32811 GAGCTTTAAGACATGGAGAACAAGCTTCTATAGATAAAATACAAGAGAGATACCCTGCTT 32752

Query 301 TCTTTGATGAAGGTAGTGGAAATTCTTCCATAAAAAAAGGTTTATATCAAGTAAGACATT 360

| ||||||||||| || |||||||||||| ||||||||||||||||||||||||||||||

Sbjct 32751 TTTTTGATGAAGGGAGCGGAAATTCTTCCGTAAAAAAAGGTTTATATCAAGTAAGACATT 32692

Query 361 ATATAGAAGAAGAATTTGATCTTGAAGAATTAGAAACACTAAAAGAAATAGATAGAGAAG 420

||||||||||||||||||||||||||||||||||||| | |||||||||||||||||||

Sbjct 32691 ATATAGAAGAAGAATTTGATCTTGAAGAATTAGAAACCTTGAAAGAAATAGATAGAGAAG 32632

Query 421 AAGCTAAACACATAGAAGCAAATAGGTTAATAGAAAAAAATAAACTTGAACTATTTGAAC 480

|||||||| || |||||||||||| |||||||||||||||||||||||||||||| |||

Sbjct 32631 AAGCTAAATACCTAGAAGCAAATAAGTTAATAGAAAAAAATAAACTTGAACTATTAGAAT 32572

Query 481 CTATAATGAATCCTGAAGAACTTAAACGTAAAAGAGAAGATTCTGAAGAACAATTTGATG 540

|| ||||||||||||||||||||||||||||||||||||||||||||||||||||||||

Sbjct 32571 TTACAATGAATCCTGAAGAACTTAAACGTAAAAGAGAAGATTCTGAAGAACAATTTGATG 32512

Query 541 AAATTGAAGCTAAAAAAGTAAAAATGAATCCTGAAGAACTTAAACGTAAAAGGGAAGATT 600

|||||||||||||||||||||||||||| |||||||||||||||||||||||||||||||

Sbjct 32511 AAATTGAAGCTAAAAAAGTAAAAATGAACCCTGAAGAACTTAAACGTAAAAGGGAAGATT 32452

Query 601 TTGA-GGAGGTTGAAAATAATCAACCTCCAACTAAAAGAGTAAAAATAAATCATAATAAC 659

|||| ||| ||||| || ||||||||| ||||||||||||||||||||||||||||||

Sbjct 32451 TTGAAGGA-GTTGATGATCATCAACCTCAAACTAAAAGAGTAAAAATAAATCATAATAAT 32393

Query 660 GATGATAATAATAACGGACAAAGTGGTATAGGCCCTTGTTCTGGTATTTCATCTGGTACT 719

||||||||||| ||||||||| ||||||||||||||||||||||||||||||||||||||

Sbjct 32392 GATGATAATAACAACGGACAAGGTGGTATAGGCCCTTGTTCTGGTATTTCATCTGGTACT 32333

Query 720 TCTTCAGAAGAAACTTCTACTAATGCGCGTAGTATAACTACTTTAATTATTTTATGATTA 779

||| ||||||||||||||||||||||||||||||||||||||||||||||||||||||||

Sbjct 32332 TCT-CAGAAGAAACTTCTACTAATGCGCGTAGTATAACTACTTTAATTATTTTATGATTA 32274

Query 780 GGTAGTAT 787

||||||||

Sbjct 32273 GGTAGTAT 32266

>ref|NW_009276969.1| Verticillium dahliae VdLs.17 supercont1.54 mitochondrial scaffold,

whole genome shotgun sequence

Length=19770

Score = 1275 bits (690), Expect = 0.0

Identities = 756/788 (96%), Gaps = 4/788 (1%)

Strand=Plus/Plus

Query 1 CAGAACTCGAACAAGATGATCATGATTCTCATGATGATTCTTTAGCTACTGACCCTGAAG 60

||||||||||||||||||||||||||||||||||||||||||||||||||||||||||||

Sbjct 13417 CAGAACTCGAACAAGATGATCATGATTCTCATGATGATTCTTTAGCTACTGACCCTGAAG 13476

Query 61 TAGACTCAGGTCTAGACAGCGATGAACAAGTTCAAAGTAGTGGATCCGAGGGAGATGAGA 120

|||||||||||||||| ||||||||||||||||||||||||||||||||||| |||||||

Sbjct 13477 TAGACTCAGGTCTAGATAGCGATGAACAAGTTCAAAGTAGTGGATCCGAGGG-GATGAGA 13535

Query 121 CTTCAGAAAATGAATCAGAAATTGAAGGTATAAATGAGGTTTCTGATTTTGAAGTTGAAG 180

|||||||||||||||||||||||||||||||||||||||||| ||||||||| |||||||

Sbjct 13536 CTTCAGAAAATGAATCAGAAATTGAAGGTATAAATGAGGTTTGTGATTTTGAGGTTGAAG 13595

Query 181 AAAGATCAGGTGATAAACTCACAACTGAAAGATTAGCTAATGATCAGACTCATCTTCTTA 240

||||||||||||||||||| | |||||||||||||||||||||||||||||||||||||

Sbjct 13596 AAAGATCAGGTGATAAACTTGCTACTGAAAGATTAGCTAATGATCAGACTCATCTTCTTA 13655

Query 241 GAGCTTTAAGACATGGAGAACAAGCTTCTATAGATAAAATACAAGAGAGATACCCTGCTT 300

||||||||||||||||||||||||||||||||||||||||||||||||||||||||||||

Sbjct 13656 GAGCTTTAAGACATGGAGAACAAGCTTCTATAGATAAAATACAAGAGAGATACCCTGCTT 13715

Query 301 TCTTTGATGAAGGTAGTGGAAATTCTTCCATAAAAAAAGGTTTATATCAAGTAAGACATT 360

| ||||||||||| || |||||||||||| ||||||||||||||||||||||||||||||

Sbjct 13716 TTTTTGATGAAGGGAGCGGAAATTCTTCCGTAAAAAAAGGTTTATATCAAGTAAGACATT 13775

Query 361 ATATAGAAGAAGAATTTGATCTTGAAGAATTAGAAACACTAAAAGAAATAGATAGAGAAG 420

||||||||||||||||||||||||||||||||||||| | |||||||||||||||||||

Sbjct 13776 ATATAGAAGAAGAATTTGATCTTGAAGAATTAGAAACCTTGAAAGAAATAGATAGAGAAG 13835

Query 421 AAGCTAAACACATAGAAGCAAATAGGTTAATAGAAAAAAATAAACTTGAACTATTTGAAC 480

|||||||| || |||||||||||| |||||||||||||||||||||||||||||| |||

Sbjct 13836 AAGCTAAATACCTAGAAGCAAATAAGTTAATAGAAAAAAATAAACTTGAACTATTAGAAT 13895

Query 481 CTATAATGAATCCTGAAGAACTTAAACGTAAAAGAGAAGATTCTGAAGAACAATTTGATG 540

|| ||||||||||||||||||||||||||||||||||||||||||||||||||||||||

Sbjct 13896 TTACAATGAATCCTGAAGAACTTAAACGTAAAAGAGAAGATTCTGAAGAACAATTTGATG 13955

Query 541 AAATTGAAGCTAAAAAAGTAAAAATGAATCCTGAAGAACTTAAACGTAAAAGGGAAGATT 600

|||||||||||||||||||||||||||| |||||||||||||||||||||||||||||||

Sbjct 13956 AAATTGAAGCTAAAAAAGTAAAAATGAACCCTGAAGAACTTAAACGTAAAAGGGAAGATT 14015

Query 601 TTGA-GGAGGTTGAAAATAATCAACCTCCAACTAAAAGAGTAAAAATAAATCATAATAAC 659

|||| ||| ||||| || ||||||||| ||||||||||||||||||||||||||||||

Sbjct 14016 TTGAAGGA-GTTGATGATCATCAACCTCAAACTAAAAGAGTAAAAATAAATCATAATAAT 14074

Query 660 GATGATAATAATAACGGACAAAGTGGTATAGGCCCTTGTTCTGGTATTTCATCTGGTACT 719

||||||||||| ||||||||| ||||||||||||||||||||||||||||||||||||||

Sbjct 14075 GATGATAATAACAACGGACAAGGTGGTATAGGCCCTTGTTCTGGTATTTCATCTGGTACT 14134

Query 720 TCTTCAGAAGAAACTTCTACTAATGCGCGTAGTATAACTACTTTAATTATTTTATGATTA 779

||||||||||||||||||||||||||||||||||||||||||||||||| ||||||||||

Sbjct 14135 TCTTCAGAAGAAACTTCTACTAATGCGCGTAGTATAACTACTTTAATTA-TTTATGATTA 14193

Query 780 GGTAGTAT 787

||||||||

Sbjct 14194 GGTAGTAT 14201

>ref|NC_008248.1| Verticillium dahliae mitochondrion, complete genome

Length=27184

Score = 1271 bits (688), Expect = 0.0

Identities = 755/788 (96%), Gaps = 2/788 (0%)

Strand=Plus/Plus

Query 1 CAGAACTCGAACAAGATGATCATGATTCTCATGATGATTCTTTAGCTACTGACCCTGAAG 60

||||||||||||||||||||||||||||||||||||||||||||||||||||||||||||

Sbjct 15557 CAGAACTCGAACAAGATGATCATGATTCTCATGATGATTCTTTAGCTACTGACCCTGAAG 15616

Query 61 TAGACTCAGGTCTAGACAGCGATGAACAAGTTCAAAGTAGTGGATCCGAGGGAGATGAGA 120

|||||||||||||||| ||||||||||||||||||||||||||||||||||| |||||||

Sbjct 15617 TAGACTCAGGTCTAGATAGCGATGAACAAGTTCAAAGTAGTGGATCCGAGGGGGATGAGA 15676

Query 121 CTTCAGAAAATGAATCAGAAATTGAAGGTATAAATGAGGTTTCTGATTTTGAAGTTGAAG 180

|||||||||||||||||||||||||||||||||||||||||| ||||||||| |||||||

Sbjct 15677 CTTCAGAAAATGAATCAGAAATTGAAGGTATAAATGAGGTTTGTGATTTTGAGGTTGAAG 15736

Query 181 AAAGATCAGGTGATAAACTCACAACTGAAAGATTAGCTAATGATCAGACTCATCTTCTTA 240

||||||||||||||||||| | |||||||||||||||||||||||||||||||||||||

Sbjct 15737 AAAGATCAGGTGATAAACTTGCTACTGAAAGATTAGCTAATGATCAGACTCATCTTCTTA 15796

Query 241 GAGCTTTAAGACATGGAGAACAAGCTTCTATAGATAAAATACAAGAGAGATACCCTGCTT 300

||||||||||||||||||||||||||||||||||||||||||||||||||||||||||||

Sbjct 15797 GAGCTTTAAGACATGGAGAACAAGCTTCTATAGATAAAATACAAGAGAGATACCCTGCTT 15856

Query 301 TCTTTGATGAAGGTAGTGGAAATTCTTCCATAAAAAAAGGTTTATATCAAGTAAGACATT 360

| ||||||||||| || |||||||||||| |||||||||||||||||||||||||| |||

Sbjct 15857 TTTTTGATGAAGGGAGCGGAAATTCTTCCGTAAAAAAAGGTTTATATCAAGTAAGAAATT 15916

Query 361 ATATAGAAGAAGAATTTGATCTTGAAGAATTAGAAACACTAAAAGAAATAGATAGAGAAG 420

||||||||||||||||||||||||||||||||||||| | |||||||||||||||||||

Sbjct 15917 ATATAGAAGAAGAATTTGATCTTGAAGAATTAGAAACCTTGAAAGAAATAGATAGAGAAG 15976

Query 421 AAGCTAAACACATAGAAGCAAATAGGTTAATAGAAAAAAATAAACTTGAACTATTTGAAC 480

|||||||| || |||||||||||| || ||||||||||||||||||||||||||| |||

Sbjct 15977 AAGCTAAATACCTAGAAGCAAATAAGTGAATAGAAAAAAATAAACTTGAACTATTAGAAT 16036

Query 481 CTATAATGAATCCTGAAGAACTTAAACGTAAAAGAGAAGATTCTGAAGAACAATTTGATG 540

|| ||||||||||||||||||||||||||||||||||||||||||||||||||||||||

Sbjct 16037 TTACAATGAATCCTGAAGAACTTAAACGTAAAAGAGAAGATTCTGAAGAACAATTTGATG 16096

Query 541 AAATTGAAGCTAAAAAAGTAAAAATGAATCCTGAAGAACTTAAACGTAAAAGGGAAGATT 600

|||||||||||||||||||||||||||| |||||||||||||||||||||||||||||||

Sbjct 16097 AAATTGAAGCTAAAAAAGTAAAAATGAACCCTGAAGAACTTAAACGTAAAAGGGAAGATT 16156

Query 601 TTGA-GGAGGTTGAAAATAATCAACCTCCAACTAAAAGAGTAAAAATAAATCATAATAAC 659

|||| ||| ||||| || ||||||||| ||||||||||||||||||||||||||||||

Sbjct 16157 TTGAAGGA-GTTGATGATCATCAACCTCAAACTAAAAGAGTAAAAATAAATCATAATAAT 16215

Query 660 GATGATAATAATAACGGACAAAGTGGTATAGGCCCTTGTTCTGGTATTTCATCTGGTACT 719

||||||||||| ||||||||| ||||||||||||||||||||||||||||||||||||||

Sbjct 16216 GATGATAATAACAACGGACAAGGTGGTATAGGCCCTTGTTCTGGTATTTCATCTGGTACT 16275

Query 720 TCTTCAGAAGAAACTTCTACTAATGCGCGTAGTATAACTACTTTAATTATTTTATGATTA 779

||||||||||||||||||||||||||||||||||||||||||||||||||||||||||||

Sbjct 16276 TCTTCAGAAGAAACTTCTACTAATGCGCGTAGTATAACTACTTTAATTATTTTATGATTA 16335

Query 780 GGTAGTAT 787

||||||||

Sbjct 16336 GGTAGTAT 16343

>ref|NW_009276924.1| Verticillium dahliae VdLs.17 supercont1.21 genomic scaffold,

whole genome shotgun sequence

Length=641803

Score = 246 bits (133), Expect = 3e-060

Identities = 186/212 (88%), Gaps = 2/212 (1%)

Strand=Plus/Plus

Query 7 TCGAACAAGATGATCATGATTCTCATGATGATTCTTTAGCTACTGACCCTGAAGTAGACT 66

|||| || |||||||||||||||||||||||||||||||||||||||||||||||| |||

Sbjct 206871 TCGACCATGATGATCATGATTCTCATGATGATTCTTTAGCTACTGACCCTGAAGTACACT 206930

Query 67 CAGGTCTAGACAGCGATGAACAAGTTCAAAGTAGTGGATCCGAGGGAGATGAGACTTCAG 126

|| ||||||| |||||||| |||||||| ||||||||||||||||| || ||||||||

Sbjct 206931 CAAGTCTAGATAGCGATGAGCAAGTTCAGAGTAGTGGATCCGAGGGGGAGCAGACTTCAC 206990

Query 127 AAAATGAATCAGAAATTGAAGGTATAAATGAGGTTTCTGATTTTGAAGTTGAAGAAAGAT 186

| | ||| ||| | |||||||||||| ||||||||| ||||||||| |||| ||||| ||

Sbjct 206991 ACAGTGAGTCATACATTGAAGGTATACATGAGGTTTGTGATTTTGAGGTTG-AGAAACAT 207049

Query 187 CAGGTGATAAACTCACAACTGAAAGATTAGCT 218

||||||| ||||| |||||||| ||| ||||

Sbjct 207050 CAGGTGACAAACTTGCAACTGAA-GATAAGCT 207080

>ref|NW_003315033.1| Verticillium albo-atrum VaMs.102 supercont1.6 genomic scaffold,

whole genome shotgun sequence

Length=2315232

Score = 80.5 bits (43), Expect = 3e-010

Identities = 49/52 (94%), Gaps = 0/52 (0%)

Strand=Plus/Minus

Query 1 CAGAACTCGAACAAGATGATCATGATTCTCATGATGATTCTTTAGCTACTGA 52

|||||||| || |||||||||||||||||||||||||||||||||||||||

Sbjct 1659624 CAGAACTCAAATCAGATGATCATGATTCTCATGATGATTCTTTAGCTACTGA 1659573

BLASTn: Refseq Representative genomes (refseq_representative_genomes)

Database: RefSeq Representative Genome Database

10,990,398 sequences; 422,001,285,913 total letters

Query= orf414

Length=789

Score E

Sequences producing significant alignments: (Bits) Value

ref|NW_009276970.1| Verticillium dahliae VdLs.17 supercont1.53 m... 1282 0.0

ref|NW_009276969.1| Verticillium dahliae VdLs.17 supercont1.54 m... 1275 0.0

ref|NW_009276924.1| Verticillium dahliae VdLs.17 supercont1.21 g... 246 2e-060

ref|NW_003315033.1| Verticillium albo-atrum VaMs.102 supercont1.... 80.5 2e-010

>ref|NW_009276970.1| Verticillium dahliae VdLs.17 supercont1.53 mitochondrial scaffold,

whole genome shotgun sequence

Length=42389

Score = 1282 bits (694), Expect = 0.0

Identities = 757/788 (96%), Gaps = 2/788 (0%)

Strand=Plus/Minus

Query 1 CAGAACTCGAACAAGATGATCATGATTCTCATGATGATTCTTTAGCTACTGACCCTGAAG 60

||||||||||||||||||||||||||||||||||||||||||||||||||||||||||||

Sbjct 6084 CAGAACTCGAACAAGATGATCATGATTCTCATGATGATTCTTTAGCTACTGACCCTGAAG 6025

Query 61 TAGACTCAGGTCTAGACAGCGATGAACAAGTTCAAAGTAGTGGATCCGAGGGAGATGAGA 120

|||||||||||||||| ||||||||||||||||||||||||||||||||||| |||||||

Sbjct 6024 TAGACTCAGGTCTAGATAGCGATGAACAAGTTCAAAGTAGTGGATCCGAGGGGGATGAGA 5965

Query 121 CTTCAGAAAATGAATCAGAAATTGAAGGTATAAATGAGGTTTCTGATTTTGAAGTTGAAG 180

|||||||||||||||||||||||||||||||||||||||||| ||||||||| |||||||

Sbjct 5964 CTTCAGAAAATGAATCAGAAATTGAAGGTATAAATGAGGTTTGTGATTTTGAGGTTGAAG 5905

Query 181 AAAGATCAGGTGATAAACTCACAACTGAAAGATTAGCTAATGATCAGACTCATCTTCTTA 240

||||||||||||||||||| | |||||||||||||||||||||||||||||||||||||

Sbjct 5904 AAAGATCAGGTGATAAACTTGCTACTGAAAGATTAGCTAATGATCAGACTCATCTTCTTA 5845

Query 241 GAGCTTTAAGACATGGAGAACAAGCTTCTATAGATAAAATACAAGAGAGATACCCTGCTT 300

||||||||||||||||||||||||||||||||||||||||||||||||||||||||||||

Sbjct 5844 GAGCTTTAAGACATGGAGAACAAGCTTCTATAGATAAAATACAAGAGAGATACCCTGCTT 5785

Query 301 TCTTTGATGAAGGTAGTGGAAATTCTTCCATAAAAAAAGGTTTATATCAAGTAAGACATT 360

| ||||||||||| || |||||||||||| ||||||||||||||||||||||||||||||

Sbjct 5784 TTTTTGATGAAGGGAGCGGAAATTCTTCCGTAAAAAAAGGTTTATATCAAGTAAGACATT 5725

Query 361 ATATAGAAGAAGAATTTGATCTTGAAGAATTAGAAACACTAAAAGAAATAGATAGAGAAG 420

||||||||||||||||||||||||||||||||||||| | |||||||||||||||||||

Sbjct 5724 ATATAGAAGAAGAATTTGATCTTGAAGAATTAGAAACCTTGAAAGAAATAGATAGAGAAG 5665

Query 421 AAGCTAAACACATAGAAGCAAATAGGTTAATAGAAAAAAATAAACTTGAACTATTTGAAC 480

|||||||| || |||||||||||| |||||||||||||||||||||||||||||| |||

Sbjct 5664 AAGCTAAATACCTAGAAGCAAATAAGTTAATAGAAAAAAATAAACTTGAACTATTAGAAT 5605

Query 481 CTATAATGAATCCTGAAGAACTTAAACGTAAAAGAGAAGATTCTGAAGAACAATTTGATG 540

|| ||||||||||||||||||||||||||||||||||||||||||||||||||||||||

Sbjct 5604 TTACAATGAATCCTGAAGAACTTAAACGTAAAAGAGAAGATTCTGAAGAACAATTTGATG 5545

Query 541 AAATTGAAGCTAAAAAAGTAAAAATGAATCCTGAAGAACTTAAACGTAAAAGGGAAGATT 600

|||||||||||||||||||||||||||| |||||||||||||||||||||||||||||||

Sbjct 5544 AAATTGAAGCTAAAAAAGTAAAAATGAACCCTGAAGAACTTAAACGTAAAAGGGAAGATT 5485

Query 601 TTGA-GGAGGTTGAAAATAATCAACCTCCAACTAAAAGAGTAAAAATAAATCATAATAAC 659

|||| ||| ||||| || ||||||||| ||||||||||||||||||||||||||||||

Sbjct 5484 TTGAAGGA-GTTGATGATCATCAACCTCAAACTAAAAGAGTAAAAATAAATCATAATAAT 5426

Query 660 GATGATAATAATAACGGACAAAGTGGTATAGGCCCTTGTTCTGGTATTTCATCTGGTACT 719

||||||||||| ||||||||| ||||||||||||||||||||||||||||||||||||||

Sbjct 5425 GATGATAATAACAACGGACAAGGTGGTATAGGCCCTTGTTCTGGTATTTCATCTGGTACT 5366

Query 720 TCTTCAGAAGAAACTTCTACTAATGCGCGTAGTATAACTACTTTAATTATTTTATGATTA 779

||||||||||||||||||||||||||||||||||||||||||||||||||||||||||||

Sbjct 5365 TCTTCAGAAGAAACTTCTACTAATGCGCGTAGTATAACTACTTTAATTATTTTATGATTA 5306

Query 780 GGTAGTAT 787

||||||||

Sbjct 5305 GGTAGTAT 5298

Score = 1275 bits (690), Expect = 0.0

Identities = 756/788 (96%), Gaps = 3/788 (0%)

Strand=Plus/Minus

Query 1 CAGAACTCGAACAAGATGATCATGATTCTCATGATGATTCTTTAGCTACTGACCCTGAAG 60

||||||||||||||||||||||||||||||||||||||||||||||||||||||||||||

Sbjct 33051 CAGAACTCGAACAAGATGATCATGATTCTCATGATGATTCTTTAGCTACTGACCCTGAAG 32992

Query 61 TAGACTCAGGTCTAGACAGCGATGAACAAGTTCAAAGTAGTGGATCCGAGGGAGATGAGA 120

|||||||||||||||| ||||||||||||||||||||||||||||||||||| |||||||

Sbjct 32991 TAGACTCAGGTCTAGATAGCGATGAACAAGTTCAAAGTAGTGGATCCGAGGGGGATGAGA 32932

Query 121 CTTCAGAAAATGAATCAGAAATTGAAGGTATAAATGAGGTTTCTGATTTTGAAGTTGAAG 180

|||||||||||||||||||||||||||||||||||||||||| ||||||||| |||||||

Sbjct 32931 CTTCAGAAAATGAATCAGAAATTGAAGGTATAAATGAGGTTTGTGATTTTGAGGTTGAAG 32872

Query 181 AAAGATCAGGTGATAAACTCACAACTGAAAGATTAGCTAATGATCAGACTCATCTTCTTA 240

||||||||||||||||||| | |||||||||||||||||||||||||||||||||||||

Sbjct 32871 AAAGATCAGGTGATAAACTTGCTACTGAAAGATTAGCTAATGATCAGACTCATCTTCTTA 32812

Query 241 GAGCTTTAAGACATGGAGAACAAGCTTCTATAGATAAAATACAAGAGAGATACCCTGCTT 300

||||||||||||||||||||||||||||||||||||||||||||||||||||||||||||

Sbjct 32811 GAGCTTTAAGACATGGAGAACAAGCTTCTATAGATAAAATACAAGAGAGATACCCTGCTT 32752

Query 301 TCTTTGATGAAGGTAGTGGAAATTCTTCCATAAAAAAAGGTTTATATCAAGTAAGACATT 360

| ||||||||||| || |||||||||||| ||||||||||||||||||||||||||||||

Sbjct 32751 TTTTTGATGAAGGGAGCGGAAATTCTTCCGTAAAAAAAGGTTTATATCAAGTAAGACATT 32692

Query 361 ATATAGAAGAAGAATTTGATCTTGAAGAATTAGAAACACTAAAAGAAATAGATAGAGAAG 420

||||||||||||||||||||||||||||||||||||| | |||||||||||||||||||

Sbjct 32691 ATATAGAAGAAGAATTTGATCTTGAAGAATTAGAAACCTTGAAAGAAATAGATAGAGAAG 32632

Query 421 AAGCTAAACACATAGAAGCAAATAGGTTAATAGAAAAAAATAAACTTGAACTATTTGAAC 480

|||||||| || |||||||||||| |||||||||||||||||||||||||||||| |||

Sbjct 32631 AAGCTAAATACCTAGAAGCAAATAAGTTAATAGAAAAAAATAAACTTGAACTATTAGAAT 32572

Query 481 CTATAATGAATCCTGAAGAACTTAAACGTAAAAGAGAAGATTCTGAAGAACAATTTGATG 540

|| ||||||||||||||||||||||||||||||||||||||||||||||||||||||||

Sbjct 32571 TTACAATGAATCCTGAAGAACTTAAACGTAAAAGAGAAGATTCTGAAGAACAATTTGATG 32512

Query 541 AAATTGAAGCTAAAAAAGTAAAAATGAATCCTGAAGAACTTAAACGTAAAAGGGAAGATT 600

|||||||||||||||||||||||||||| |||||||||||||||||||||||||||||||

Sbjct 32511 AAATTGAAGCTAAAAAAGTAAAAATGAACCCTGAAGAACTTAAACGTAAAAGGGAAGATT 32452

Query 601 TTGA-GGAGGTTGAAAATAATCAACCTCCAACTAAAAGAGTAAAAATAAATCATAATAAC 659

|||| ||| ||||| || ||||||||| ||||||||||||||||||||||||||||||

Sbjct 32451 TTGAAGGA-GTTGATGATCATCAACCTCAAACTAAAAGAGTAAAAATAAATCATAATAAT 32393

Query 660 GATGATAATAATAACGGACAAAGTGGTATAGGCCCTTGTTCTGGTATTTCATCTGGTACT 719

||||||||||| ||||||||| ||||||||||||||||||||||||||||||||||||||

Sbjct 32392 GATGATAATAACAACGGACAAGGTGGTATAGGCCCTTGTTCTGGTATTTCATCTGGTACT 32333

Query 720 TCTTCAGAAGAAACTTCTACTAATGCGCGTAGTATAACTACTTTAATTATTTTATGATTA 779

||| ||||||||||||||||||||||||||||||||||||||||||||||||||||||||

Sbjct 32332 TCT-CAGAAGAAACTTCTACTAATGCGCGTAGTATAACTACTTTAATTATTTTATGATTA 32274

Query 780 GGTAGTAT 787

||||||||

Sbjct 32273 GGTAGTAT 32266

>ref|NW_009276969.1| Verticillium dahliae VdLs.17 supercont1.54 mitochondrial scaffold,

whole genome shotgun sequence

Length=19770

Score = 1275 bits (690), Expect = 0.0

Identities = 756/788 (96%), Gaps = 4/788 (1%)

Strand=Plus/Plus

Query 1 CAGAACTCGAACAAGATGATCATGATTCTCATGATGATTCTTTAGCTACTGACCCTGAAG 60

||||||||||||||||||||||||||||||||||||||||||||||||||||||||||||

Sbjct 13417 CAGAACTCGAACAAGATGATCATGATTCTCATGATGATTCTTTAGCTACTGACCCTGAAG 13476

Query 61 TAGACTCAGGTCTAGACAGCGATGAACAAGTTCAAAGTAGTGGATCCGAGGGAGATGAGA 120

|||||||||||||||| ||||||||||||||||||||||||||||||||||| |||||||

Sbjct 13477 TAGACTCAGGTCTAGATAGCGATGAACAAGTTCAAAGTAGTGGATCCGAGGG-GATGAGA 13535

Query 121 CTTCAGAAAATGAATCAGAAATTGAAGGTATAAATGAGGTTTCTGATTTTGAAGTTGAAG 180

|||||||||||||||||||||||||||||||||||||||||| ||||||||| |||||||

Sbjct 13536 CTTCAGAAAATGAATCAGAAATTGAAGGTATAAATGAGGTTTGTGATTTTGAGGTTGAAG 13595

Query 181 AAAGATCAGGTGATAAACTCACAACTGAAAGATTAGCTAATGATCAGACTCATCTTCTTA 240

||||||||||||||||||| | |||||||||||||||||||||||||||||||||||||

Sbjct 13596 AAAGATCAGGTGATAAACTTGCTACTGAAAGATTAGCTAATGATCAGACTCATCTTCTTA 13655

Query 241 GAGCTTTAAGACATGGAGAACAAGCTTCTATAGATAAAATACAAGAGAGATACCCTGCTT 300

||||||||||||||||||||||||||||||||||||||||||||||||||||||||||||

Sbjct 13656 GAGCTTTAAGACATGGAGAACAAGCTTCTATAGATAAAATACAAGAGAGATACCCTGCTT 13715

Query 301 TCTTTGATGAAGGTAGTGGAAATTCTTCCATAAAAAAAGGTTTATATCAAGTAAGACATT 360

| ||||||||||| || |||||||||||| ||||||||||||||||||||||||||||||

Sbjct 13716 TTTTTGATGAAGGGAGCGGAAATTCTTCCGTAAAAAAAGGTTTATATCAAGTAAGACATT 13775

Query 361 ATATAGAAGAAGAATTTGATCTTGAAGAATTAGAAACACTAAAAGAAATAGATAGAGAAG 420

||||||||||||||||||||||||||||||||||||| | |||||||||||||||||||

Sbjct 13776 ATATAGAAGAAGAATTTGATCTTGAAGAATTAGAAACCTTGAAAGAAATAGATAGAGAAG 13835

Query 421 AAGCTAAACACATAGAAGCAAATAGGTTAATAGAAAAAAATAAACTTGAACTATTTGAAC 480

|||||||| || |||||||||||| |||||||||||||||||||||||||||||| |||

Sbjct 13836 AAGCTAAATACCTAGAAGCAAATAAGTTAATAGAAAAAAATAAACTTGAACTATTAGAAT 13895

Query 481 CTATAATGAATCCTGAAGAACTTAAACGTAAAAGAGAAGATTCTGAAGAACAATTTGATG 540

|| ||||||||||||||||||||||||||||||||||||||||||||||||||||||||

Sbjct 13896 TTACAATGAATCCTGAAGAACTTAAACGTAAAAGAGAAGATTCTGAAGAACAATTTGATG 13955

Query 541 AAATTGAAGCTAAAAAAGTAAAAATGAATCCTGAAGAACTTAAACGTAAAAGGGAAGATT 600

|||||||||||||||||||||||||||| |||||||||||||||||||||||||||||||

Sbjct 13956 AAATTGAAGCTAAAAAAGTAAAAATGAACCCTGAAGAACTTAAACGTAAAAGGGAAGATT 14015

Query 601 TTGA-GGAGGTTGAAAATAATCAACCTCCAACTAAAAGAGTAAAAATAAATCATAATAAC 659

|||| ||| ||||| || ||||||||| ||||||||||||||||||||||||||||||

Sbjct 14016 TTGAAGGA-GTTGATGATCATCAACCTCAAACTAAAAGAGTAAAAATAAATCATAATAAT 14074

Query 660 GATGATAATAATAACGGACAAAGTGGTATAGGCCCTTGTTCTGGTATTTCATCTGGTACT 719

||||||||||| ||||||||| ||||||||||||||||||||||||||||||||||||||

Sbjct 14075 GATGATAATAACAACGGACAAGGTGGTATAGGCCCTTGTTCTGGTATTTCATCTGGTACT 14134

Query 720 TCTTCAGAAGAAACTTCTACTAATGCGCGTAGTATAACTACTTTAATTATTTTATGATTA 779

||||||||||||||||||||||||||||||||||||||||||||||||| ||||||||||

Sbjct 14135 TCTTCAGAAGAAACTTCTACTAATGCGCGTAGTATAACTACTTTAATTA-TTTATGATTA 14193

Query 780 GGTAGTAT 787

||||||||

Sbjct 14194 GGTAGTAT 14201

>ref|NW_009276924.1| Verticillium dahliae VdLs.17 supercont1.21 genomic scaffold,

whole genome shotgun sequence

Length=641803

Score = 246 bits (133), Expect = 2e-060

Identities = 186/212 (88%), Gaps = 2/212 (1%)

Strand=Plus/Plus

Query 7 TCGAACAAGATGATCATGATTCTCATGATGATTCTTTAGCTACTGACCCTGAAGTAGACT 66

|||| || |||||||||||||||||||||||||||||||||||||||||||||||| |||

Sbjct 206871 TCGACCATGATGATCATGATTCTCATGATGATTCTTTAGCTACTGACCCTGAAGTACACT 206930

Query 67 CAGGTCTAGACAGCGATGAACAAGTTCAAAGTAGTGGATCCGAGGGAGATGAGACTTCAG 126

|| ||||||| |||||||| |||||||| ||||||||||||||||| || ||||||||

Sbjct 206931 CAAGTCTAGATAGCGATGAGCAAGTTCAGAGTAGTGGATCCGAGGGGGAGCAGACTTCAC 206990

Query 127 AAAATGAATCAGAAATTGAAGGTATAAATGAGGTTTCTGATTTTGAAGTTGAAGAAAGAT 186

| | ||| ||| | |||||||||||| ||||||||| ||||||||| |||| ||||| ||

Sbjct 206991 ACAGTGAGTCATACATTGAAGGTATACATGAGGTTTGTGATTTTGAGGTTG-AGAAACAT 207049

Query 187 CAGGTGATAAACTCACAACTGAAAGATTAGCT 218

||||||| ||||| |||||||| ||| ||||

Sbjct 207050 CAGGTGACAAACTTGCAACTGAA-GATAAGCT 207080

>ref|NW_003315033.1| Verticillium albo-atrum VaMs.102 supercont1.6 genomic scaffold,

whole genome shotgun sequence

Length=2315232

Score = 80.5 bits (43), Expect = 2e-010

Identities = 49/52 (94%), Gaps = 0/52 (0%)

Strand=Plus/Minus

Query 1 CAGAACTCGAACAAGATGATCATGATTCTCATGATGATTCTTTAGCTACTGA 52

|||||||| || |||||||||||||||||||||||||||||||||||||||

Sbjct 1659624 CAGAACTCAAATCAGATGATCATGATTCTCATGATGATTCTTTAGCTACTGA 1659573

BLASTn: NCBI Genomes (chromsome)

Database: NCBI Chromosome Sequences

86,835 sequences; 362,426,053,891 total letters

Query= orf414

Length=789

Score E

Sequences producing significant alignments: (Bits) Value

ref|NC_008248.1| Verticillium dahliae mitochondrion, complete ge... 1271 0.0

ref|NW_003315033.1| Verticillium albo-atrum VaMs.102 supercont1.... 80.5 2e-010

>ref|NC_008248.1| Verticillium dahliae mitochondrion, complete genome

Length=27184

Score = 1271 bits (688), Expect = 0.0

Identities = 755/788 (96%), Gaps = 2/788 (0%)

Strand=Plus/Plus

Query 1 CAGAACTCGAACAAGATGATCATGATTCTCATGATGATTCTTTAGCTACTGACCCTGAAG 60

||||||||||||||||||||||||||||||||||||||||||||||||||||||||||||

Sbjct 15557 CAGAACTCGAACAAGATGATCATGATTCTCATGATGATTCTTTAGCTACTGACCCTGAAG 15616

Query 61 TAGACTCAGGTCTAGACAGCGATGAACAAGTTCAAAGTAGTGGATCCGAGGGAGATGAGA 120

|||||||||||||||| ||||||||||||||||||||||||||||||||||| |||||||

Sbjct 15617 TAGACTCAGGTCTAGATAGCGATGAACAAGTTCAAAGTAGTGGATCCGAGGGGGATGAGA 15676

Query 121 CTTCAGAAAATGAATCAGAAATTGAAGGTATAAATGAGGTTTCTGATTTTGAAGTTGAAG 180

|||||||||||||||||||||||||||||||||||||||||| ||||||||| |||||||

Sbjct 15677 CTTCAGAAAATGAATCAGAAATTGAAGGTATAAATGAGGTTTGTGATTTTGAGGTTGAAG 15736

Query 181 AAAGATCAGGTGATAAACTCACAACTGAAAGATTAGCTAATGATCAGACTCATCTTCTTA 240

||||||||||||||||||| | |||||||||||||||||||||||||||||||||||||

Sbjct 15737 AAAGATCAGGTGATAAACTTGCTACTGAAAGATTAGCTAATGATCAGACTCATCTTCTTA 15796

Query 241 GAGCTTTAAGACATGGAGAACAAGCTTCTATAGATAAAATACAAGAGAGATACCCTGCTT 300

||||||||||||||||||||||||||||||||||||||||||||||||||||||||||||

Sbjct 15797 GAGCTTTAAGACATGGAGAACAAGCTTCTATAGATAAAATACAAGAGAGATACCCTGCTT 15856

Query 301 TCTTTGATGAAGGTAGTGGAAATTCTTCCATAAAAAAAGGTTTATATCAAGTAAGACATT 360

| ||||||||||| || |||||||||||| |||||||||||||||||||||||||| |||

Sbjct 15857 TTTTTGATGAAGGGAGCGGAAATTCTTCCGTAAAAAAAGGTTTATATCAAGTAAGAAATT 15916

Query 361 ATATAGAAGAAGAATTTGATCTTGAAGAATTAGAAACACTAAAAGAAATAGATAGAGAAG 420

||||||||||||||||||||||||||||||||||||| | |||||||||||||||||||

Sbjct 15917 ATATAGAAGAAGAATTTGATCTTGAAGAATTAGAAACCTTGAAAGAAATAGATAGAGAAG 15976

Query 421 AAGCTAAACACATAGAAGCAAATAGGTTAATAGAAAAAAATAAACTTGAACTATTTGAAC 480

|||||||| || |||||||||||| || ||||||||||||||||||||||||||| |||

Sbjct 15977 AAGCTAAATACCTAGAAGCAAATAAGTGAATAGAAAAAAATAAACTTGAACTATTAGAAT 16036

Query 481 CTATAATGAATCCTGAAGAACTTAAACGTAAAAGAGAAGATTCTGAAGAACAATTTGATG 540

|| ||||||||||||||||||||||||||||||||||||||||||||||||||||||||

Sbjct 16037 TTACAATGAATCCTGAAGAACTTAAACGTAAAAGAGAAGATTCTGAAGAACAATTTGATG 16096

Query 541 AAATTGAAGCTAAAAAAGTAAAAATGAATCCTGAAGAACTTAAACGTAAAAGGGAAGATT 600

|||||||||||||||||||||||||||| |||||||||||||||||||||||||||||||

Sbjct 16097 AAATTGAAGCTAAAAAAGTAAAAATGAACCCTGAAGAACTTAAACGTAAAAGGGAAGATT 16156

Query 601 TTGA-GGAGGTTGAAAATAATCAACCTCCAACTAAAAGAGTAAAAATAAATCATAATAAC 659

|||| ||| ||||| || ||||||||| ||||||||||||||||||||||||||||||

Sbjct 16157 TTGAAGGA-GTTGATGATCATCAACCTCAAACTAAAAGAGTAAAAATAAATCATAATAAT 16215

Query 660 GATGATAATAATAACGGACAAAGTGGTATAGGCCCTTGTTCTGGTATTTCATCTGGTACT 719

||||||||||| ||||||||| ||||||||||||||||||||||||||||||||||||||

Sbjct 16216 GATGATAATAACAACGGACAAGGTGGTATAGGCCCTTGTTCTGGTATTTCATCTGGTACT 16275

Query 720 TCTTCAGAAGAAACTTCTACTAATGCGCGTAGTATAACTACTTTAATTATTTTATGATTA 779

||||||||||||||||||||||||||||||||||||||||||||||||||||||||||||

Sbjct 16276 TCTTCAGAAGAAACTTCTACTAATGCGCGTAGTATAACTACTTTAATTATTTTATGATTA 16335

Query 780 GGTAGTAT 787

||||||||

Sbjct 16336 GGTAGTAT 16343

>ref|NW_003315033.1| Verticillium albo-atrum VaMs.102 supercont1.6 genomic scaffold,

whole genome shotgun sequence

Length=2315232

Score = 80.5 bits (43), Expect = 2e-010

Identities = 49/52 (94%), Gaps = 0/52 (0%)

Strand=Plus/Minus

Query 1 CAGAACTCGAACAAGATGATCATGATTCTCATGATGATTCTTTAGCTACTGA 52

|||||||| || |||||||||||||||||||||||||||||||||||||||

Sbjct 1659624 CAGAACTCAAATCAGATGATCATGATTCTCATGATGATTCTTTAGCTACTGA 1659573

BLASTn: Expressed sequence tags (est)

Database: Database of GenBank+EMBL+DDBJ sequences from EST Divisions

76,041,847 sequences; 42,369,095,621 total letters

Query= orf414

Length=789

Score E

Sequences producing significant alignments: (Bits) Value

gb|GR299985.1| G969P31RA11.T0 Phytophthora infestans T30-4 NO... 686 0.0

ALIGNMENTS

>gb|GR299985.1| G969P31RA11.T0 Phytophthora infestans T30-4 NORMALIZED Phytophthora

infestans cDNA, mRNA sequence.

Length=705

Score = 686 bits (760), Expect = 0.0

Identities = 416/440 (95%), Gaps = 0/440 (0%)

Strand=Plus/Minus

Query 348 CAAGTAAGACATTATATAGAAGAAGAATTTGATCTTGAAGAATTAGAAACACTAAAAGAA 407

|||||||| |||||||||||||||||||||||||||||||| |||||||| | ||||||

Sbjct 705 CAAGTAAGCCATTATATAGAAGAAGAATTTGATCTTGAAGAGTTAGAAACCTTGAAAGAA 646

Query 408 ATAGATAGAGAAGAAGCTAAACACATAGAAGCAAATAGGTTAATAGAAAAAAATAAACTT 467

||||||||||||||||||||| || |||||||||||| ||||||||||||||||||||||

Sbjct 645 ATAGATAGAGAAGAAGCTAAATACCTAGAAGCAAATAAGTTAATAGAAAAAAATAAACTT 586

Query 468 GAACTATTTGAACCTATAATGAATCCTGAAGAACTTAAACGTAAAAGAGAAGATTCTGAA 527

|||||||| ||| || |||||||||||||||||||||||||||||||||||||||||||

Sbjct 585 GAACTATTAGAATTTACAATGAATCCTGAAGAACTTAAACGTAAAAGAGAAGATTCTGAA 526

Query 528 GAACAATTTGATGAAATTGAAGCTAAAAAAGTAAAAATGAATCCTGAAGAACTTAAACGT 587

||||||||||||||||||||||||||||||||||||||||| ||||||||||||||||||

Sbjct 525 GAACAATTTGATGAAATTGAAGCTAAAAAAGTAAAAATGAACCCTGAAGAACTTAAACGT 466

Query 588 AAAAGGGAAGATTTTGAGGAGGTTGAAAATAATCAACCTCCAACTAAAAGAGTAAAAATA 647

||||||||||||||||| | ||||| || ||||||||| |||||||||||||||||||

Sbjct 465 AAAAGGGAAGATTTTGAAGGAGTTGATGATCATCAACCTCAAACTAAAAGAGTAAAAATA 406

Query 648 AATCATAATAACGATGATAATAATAACGGACAAAGTGGTATAGGCCCTTGTTCTGGTATT 707

||||||||||| ||||||||||| ||||||||| ||||||||||||||||||||||||||

Sbjct 405 AATCATAATAATGATGATAATAACAACGGACAAGGTGGTATAGGCCCTTGTTCTGGTATT 346

Query 708 TCATCTGGTACTTCTTCAGAAGAAACTTCTACTAATGCGCGTAGTATAACTACTTTAATT 767

|||||||||||||||||||||||||||||||||||||||||||||||| |||||||||||

Sbjct 345 TCATCTGGTACTTCTTCAGAAGAAACTTCTACTAATGCGCGTAGTATACCTACTTTAATT 286

Query 768 ATTTTATGATTAGGTAGTAT 787

||||||||||||||||||||

Sbjct 285 ATTTTATGATTAGGTAGTAT 266

BLASTn: Genomic survey sequences (gss)

No significant similarity found.

BLASTn: High throughput genomic sequences (HTGS)

No significant similarity found.

BLASTn: Transcriptome Shotgun Assembly (TSA) sequences (tsa_all)

No significant similarity found.

BLASTx: Non-redundant protein sequences (nr)

No significant similarity found.
