# Supplementary material for: Complete mitochondrial genome of the Verticillium-wilt causing plant pathogen Verticillium nonalfalfae
Source: PLoS One. 2016 Feb 3;11(2):e0148525. doi: 10.1371/journal.pone.0148525 (PMC4739603; doi:10.1371/journal.pone.0148525)
Supplement: S1 Table — (DOC) [file pone.0148525.s004.doc]

**Supplementary Table 1: Codon usage statistics**

| **Codon** | **Amino Acid Residue** | **Fraction** | **Number** |
| --- | --- | --- | --- |
| GCA | A | 0.224 | 46 |
| GCC | A | 0.112 | 23 |
| GCG | A | 0.034 | 7 |
| GCU | A | 0.629 | 129 |
| UGC | C | 0.34 | 54 |
| UGU | C | 0.66 | 105 |
| GAC | D | 0.318 | 41 |
| GAU | D | 0.682 | 88 |
| GAA | E | 0.737 | 98 |
| GAG | E | 0.263 | 35 |
| UUC | F | 0.27 | 148 |
| UUU | F | 0.73 | 401 |
| GGA | G | 0.411 | 104 |
| GGC | G | 0.055 | 14 |
| GGG | G | 0.111 | 28 |
| GGU | G | 0.423 | 107 |
| CAC | H | 0.301 | 37 |
| CAU | H | 0.699 | 86 |
| AUA | I | 0.39 | 311 |
| AUC | I | 0.129 | 103 |
| AUU | I | 0.481 | 384 |
| AAA | K | 0.685 | 231 |
| AAG | K | 0.315 | 106 |
| CUA | L | 0.083 | 66 |
| CUC | L | 0.033 | 26 |
| CUG | L | 0.052 | 41 |
| CUU | L | 0.163 | 129 |
| UUA | L | 0.585 | 463 |
| UUG | L | 0.083 | 66 |
| AUG | M | 1 | 124 |
| AAC | N | 0.285 | 112 |
| AAU | N | 0.715 | 281 |
| CCA | P | 0.244 | 41 |
| CCC | P | 0.131 | 22 |
| CCG | P | 0.06 | 10 |
| CCU | P | 0.565 | 95 |
| CAA | Q | 0.7 | 91 |
| CAG | Q | 0.3 | 39 |
| AGA | R | 0.433 | 120 |
| AGG | R | 0.296 | 82 |
| CGA | R | 0.072 | 20 |
| CGC | R | 0.065 | 18 |
| CGG | R | 0.058 | 16 |
| CGU | R | 0.076 | 21 |
| AGC | S | 0.149 | 79 |
| AGU | S | 0.312 | 165 |
| UCA | S | 0.172 | 91 |
| UCC | S | 0.076 | 40 |
| UCG | S | 0.036 | 19 |
| UCU | S | 0.255 | 135 |
| ACA | T | 0.416 | 127 |
| ACC | T | 0.144 | 44 |
| ACG | T | 0.066 | 20 |
| ACU | T | 0.374 | 114 |
| GUA | V | 0.451 | 144 |
| GUC | V | 0.069 | 22 |
| GUG | V | 0.091 | 29 |
| GUU | V | 0.389 | 124 |
| UGG | W | 1 | 51 |
| UAC | Y | 0.288 | 132 |
| UAU | Y | 0.712 | 327 |
| UAA | * | 0.463 | 189 |
| UAG | * | 0.26 | 106 |
| UGA | * | 0.277 | 113 |
